# *Glycine max* Homologs of *DOESN'T MAKE INFECTIONS 1, 2*, and *3* Function to Impair *Heterodera glycines* Parasitism While Also Regulating Mitogen Activated Protein Kinase Expression

**DOI:** 10.3389/fpls.2022.842597

**Published:** 2022-05-04

**Authors:** Rishi Khatri, Shankar R. Pant, Keshav Sharma, Prakash M. Niraula, Bisho R. Lawaju, Kathy S. Lawrence, Nadim W. Alkharouf, Vincent P. Klink

**Affiliations:** ^1^Department of Biological Sciences, Mississippi State University, Starkville, MS, United States; ^2^Department of Biochemistry, Molecular Biology, Entomology and Plant Pathology, Mississippi State University, Starkville, MS, United States; ^3^Department of Entomology and Plant Pathology, Auburn University, Auburn, AL, United States; ^4^Department of Computer and Information Sciences, Towson University, Towson, MD, United States; ^5^USDA ARS NEA BARC Molecular Plant Pathology Laboratory, Beltsville, MD, United States; ^6^Center for Computational Sciences High Performance Computing Collaboratory, Mississippi State University, Starkville, MS, United States

**Keywords:** plant parasitic nematode, pathogen recognition receptor (PRR), effector triggered immunity (ETI), pathogen associated molecular pattern (PAMP), PAMP triggered immunity (PTI), *Glycine max*, common symbiosis pathway (CSP), *DOESN'T MAKE INFECTIONS (DMI)*

## Abstract

*Glycine max* root cells developing into syncytia through the parasitic activities of the pathogenic nematode *Heterodera glycines* underwent isolation by laser microdissection (LM). Microarray analyses have identified the expression of a *G. max DOESN'T MAKE INFECTIONS3* (*DMI3*) homolog in syncytia undergoing parasitism but during a defense response. *DMI3* encodes part of the common symbiosis pathway (CSP) involving *DMI1, DMI2*, and other CSP genes. The identified *DMI* gene expression, and symbiosis role, suggests the possible existence of commonalities between symbiosis and defense. *G. max* has 3 *DMI1*, 12 *DMI2*, and 2 *DMI3* paralogs. LM-assisted gene expression experiments of isolated syncytia under further examination here show *G. max DMI1-3, DMI2-7*, and *DMI3-2* expression occurring during the defense response in the *H. glycines*-resistant genotypes *G*.*max*_[Peking/PI548402]_ and *G*.*max*_[PI88788]_ indicating a broad and consistent level of expression of the genes. Transgenic overexpression (OE) of *G. max DMI1-3, DMI2-7*, and *DMI3-2* impairs *H. glycines* parasitism. RNA interference (RNAi) of *G. max DMI1-3, DMI2-7*, and *DMI3-2* increases *H. glycines* parasitism. The combined opposite outcomes reveal a defense function for these genes. Prior functional transgenic analyses of the 32-member *G. max mitogen activated protein kinase* (*MAPK*) gene family has determined that 9 of them act in the defense response to *H. glycines* parasitism, referred to as defense *MAPK*s. RNA-seq analyses of root RNA isolated from the 9 *G. max* defense *MAPK*s undergoing OE or RNAi reveal they alter the relative transcript abundances (RTAs) of specific *DMI1, DMI2*, and *DMI3* paralogs. In contrast, transgenically-manipulated *DMI1-3, DMI2-7*, and *DMI3-2* expression influences *MAPK3-1* and *MAPK3-2* RTAs under certain circumstances. The results show *G. max* homologs of the CSP, and defense pathway are linked, apparently involving co-regulated gene expression.

## Introduction

Plants are sessile organisms that respond to biotic and abiotic conditions, accordingly, to the best of their capability. In some instances, plants undergo symbiotic interactions which facilitate the improvement of their existence and fitness due to the availability of needed materials (Stanley et al., 1993). Plants also undergo pathogenic interactions which impair their ability, and detrimentally affect their fitness (Traw et al., 2007). Therefore, the processes are contrasting in nature (Lewin, 1982). Furthermore, plants can undergo these contrasting processes simultaneously, leading to a disruption of normal cell biological and physiological activities (Barker et al., 1971, 1972; Lehman et al., 1971; Hussey and Barker, 1976; Huang and Barker, 1983; Ko et al., 1984). In these instances, plants may have to choose between symbiosis and defense reactions to varying degrees, considering both processes involve the invasion of plant tissue and interaction with specific cell types, leading to impaired symbiosis at the expense of the successful pathogen. Plants even make choices between different types of symbioses that are sustained in their tissue (Bethlenfalvay et al., 1985, 1987; Hohnjec et al., 2005). These results indicate there are contrasting ways in which the underlying genetic programs function during these different processes while also being shared as important actors. Such considerations are important not only from a metabolite assimilation standpoint, but even more broadly as it relates to climate change. For example, the production of commercial synthetic nitrogen-containing fertilizers consumes 1–2% of the earth's used energy each year while also being the leading source of CO_2_ generation, contributing between 1 and 2% of worldwide emissions (Boerner, 2019). Furthermore, to combat pathogens globally, approximately 3 billion kilograms (kg) of pesticides are applied annually, costing nearly $40 billion (Pimentel, 2005; Sharma et al., 2019). Knowledge of genetic pathways functioning in both processes, and with the capability of being fine-tuned to function more effectively, is of urgent need (Ried et al., 2014; Saha et al., 2014).

An important model used to understand symbioses is *Glycine max* (soybean), undergoing such relationships with arbuscular mycorrhizal (AM) fungi as well as rhizobia bacteria that lead to the production of nodules (Bethlenfalvay et al., 1985, 1987; van Kessel et al., 1985). The AM relationship, occurring within >80% of land plants, and nodulation occurring in the Fabaceae, represent the two most important symbiotic interactions that happen between the plant root and microbes (Zhu et al., 2006). AM associations are the more ancient form, first evolving around 460 million years ago (mya) in all land plant lineages and are believed to have allowed their ancestors to colonize land (Remy et al., 1994; Redecker et al., 2000; Heckman et al., 2001; Brundrett, 2002). Nodule-forming relationships are the less ancient form that evolved ~60–70 mya in angiosperms in the Fabaceae (legumes) (Doyle and Luckow, 2003). AM fungi enhance nutrient availability, mainly inorganic phosphate, to the plants whereas legume plants form an intricate symbiotic relationship with specific soil bacteria (i.e., rhizobia) forming specialized structures called nodules which fix atmospheric nitrogen for their host (Zhu et al., 2006; Ferguson et al., 2010). In AM and nodule-forming symbiosis the microbe colonizes the plant tissue. The plant permits their entry and establishment of the microorganism so it can gain from their activities. Experiments show the AM and nodulation processes that permit microorganism (symbiont) entry and maintenance are linked genetically, revealing the molecular components are equally ancient even though they provide different beneficial metabolites (Catoira et al., 2000; Ané et al., 2002, 2004; Zhu et al., 2006; Wang et al., 2010). These observations allow a generalization of the molecular nature of symbiosis as it relates to plant defense.

About half of the non-nodulating legume mutants isolated so far are also defective in the AM symbiosis, implying that the wild-type copies of those genes are required for both processes (Catoira et al., 2000; Ané et al., 2002, 2004). The signal transduction pathway mediated by those genes is denoted as the common symbiosis pathway (CSP) (Kouchi et al., 2010). The range of symbiosis-defective phenotypes of the CSP genes leads to their grouping into two categories; one is positioned upstream of divalent calcium (Ca^2+^) spiking (upstream genes) and the other is positioned downstream of Ca^2+^ spiking (downstream genes), which is a central physiological reaction in the CSP (Ehrhardt et al., 1996; Miwa et al., 2006). Following the perception of nodulation (nod) factors (NFs) through Lysin motif receptor-like kinases (LysM-RLKs), biphasic Ca^2+^ signaling is induced in root hair cells (i.e., a rapid influx of Ca^2+^ into the root hair cells) and then the occurrence of a periodical oscillation of cytosolic Ca^2+^ concentrations at the perinuclear region (i.e., Ca^2+^ spiking). Ca^2+^ spiking is also induced in response to AM infection, critical for AM symbiosis as well as nodule symbiosis (Kosuta et al., 2008).

Study of the CSP in model legumes has led to the identification of three genes designated as *DOESN'T MAKE INFECTIONS1, 2*, and *3* (*DMI1-3*) (Catoira et al., 2000; Ané et al., 2002, 2004). *DMI* genes control the NF signaling pathway leading to nodulation, and are required for formation of mycorrhiza, indicating that the symbiotic signaling pathway activated by both the rhizobial and fungal symbionts share common steps (Oláh et al., 2005).

*DMI1* encodes a putative cation channel protein that is localized to the nuclear periphery (Riely et al., 2007). The *Medicago truncatula* (alfalfa) *Mt-DMI1*, and *Pisum sativum* (pea) *Ps-DMI1/SYM8* are putative orthologs of *L. japonicus LJ-POLLUX*, and *Lj-CASTOR* (Zhu et al., 2006; Edwards et al., 2007). The DMI1 (*M. truncatula*) and DMI1*/*SYM8 (*P. sativum*) proteins have the capacity to compensate for the loss of both CASTOR and POLLUX ion channels in *L. japonicus* in both AM and nodule symbioses (Venkateshwaran et al., 2012). *Lj-*CASTOR and *Lj-*POLLUX are non-selective ion channels with a preference for K^+^ over anions (Charpentier et al., 2008). Along with these physiological changes is the involvement of Ca^2+^. There appears to be 2 different Ca^2+^-involved processes functioning early during the symbiotic relationship. One process occurs at the root hair tip and is the generation of a Ca^2+^ gradient that is important for infection thread development while the second process involves the generation of Ca^2+^ spikes of in the nuclear region (Cardenas et al., 1999; Shaw and Long, 2003). Interestingly, *dmi1* mutants interfere with the generation of Ca^2+^ spikes (Ehrhardt et al., 1996; Wais et al., 2000; Shaw and Long, 2003; Lévy et al., 2004).

*DMI2* exists in other plant systems as the symbiosis (*SYM*) *nodulation receptor kinase* (*NORK*), and *Symbiosis receptor-like kinase* (*SYMRK*) genes. *DMI2* orthologs in *P. sativum, M. truncatula*, and *L. japonicus*, respectively, include *PsSYM19/Ms-NORK/Lj-SYMRK* that encode receptor-like kinases with leucine-rich-repeat (LRR) domains in the predicted extracellular region and possibly transmit the NF signal to the nuclear localized ion channel DMI1 (Ané et al., 2002; Endre et al., 2002; Stracke et al., 2002; Limpens et al., 2005; Riely et al., 2007; Smit et al., 2007). DMI2 is indispensable for AM- and plant-*Frankia* symbioses, and mutations in *DMI2* lead to the abortion of rhizobia infection at a very early stage (Endre et al., 2002; Stracke et al., 2002; Gherbi et al., 2008). The DMI2 protein contains an intracellular kinase domain, a transmembrane domain, and the extracellular portion, including a region with LRRs and a malectin-like domain (MLD) (Pan et al., 2018). Overexpressing the full-length *SYMRK*/*DMI2* or the intracellular kinase domain of *SYMRK*/*DMI2* results in the spontaneous nodule formation even in the absence of rhizobia (Ried et al., 2014; Saha et al., 2014). Like *dmi1, dmi2* mutants interfere with the generation of Ca^2+^ spikes (Ehrhardt et al., 1996; Wais et al., 2000; Shaw and Long, 2003; Lévy et al., 2004).

*Mt-DMI3* and its ortholog *Ps-SYM9* encode proteins with strong similarity to Ca (2+)/calmodulin-dependent protein kinase (CCaMK) (Lévy et al., 2004; Mitra et al., 2004). DMI3 plays a role downstream of the generation of Ca^2+^ spikes and is hypothesized to translate the information encoded in the Ca^2+^ spikes into one or more phosphorylation events (Ehrhardt et al., 1996; Wais et al., 2000; Lévy et al., 2004; Mitra et al., 2004). DMI3 appears to decode and transmit the information DMI3 encoded in the Ca^2+^ spikes but does not generate them since *dmi3* mutants have no effect on Ca^2+^ spiking (Ehrhardt et al., 1996; Wais et al., 2000; Lévy et al., 2004). This observation contrasts with those made for *dmi1* and *dmi2* mutants which perturb Ca^2+^ spiking (Ehrhardt et al., 1996; Cardenas et al., 1999; Wais et al., 2000; Shaw and Long, 2003). Furthermore, *dmi3* mutants exhibit increased sensitivity to NFs indicating signal transduction occurs at or downstream of DMI3 (Oldroyd et al., 2001; Shaw and Long, 2003). The functional analyses reveal the central regulatory position of CCaMK in connecting the infection and organogenetic pathways in *L. japonicus*. Additionally, components of the CSP upstream of Ca^2+^ spiking are only required for activation of CCaMK (Horváth et al., 2011). *Mt-*DMI1 and *Mt-*DMI2, acting upstream of Ca^2+^ spiking, suggests that Ca^2+^ is a component of the NF signal-transduction pathway (Oldroyd and Downie, 2004). In contrast, *Mt-*DMI3 lying downstream of Ca^2+^ spiking suggests for its possible role in perceiving the Ca^2+^ signal, decoding, and transducing the signal into an output response (Oldroyd and Downie, 2004). The absence of an *Mt-DMI3* ortholog in *Arabidopsis thaliana* may explain why it cannot establish symbiosis with AM fungi (Zhu et al., 2006). In addition to promoting downstream gene expression, DMI3 negatively regulates upstream signaling events, as *dmi3* mutants show an increased sensitivity for Ca^2+^ spiking in response to NFs and altered transcription (Oldroyd et al., 2001; Czaja et al., 2012).

AM and nodule symbioses are under competition by pathogenic organisms that can detrimentally affect their development (Winkler et al., 1994; Kennedy et al., 1999; Todd et al., 2001). One of the best examples of these detrimental relationships is with endoparasitic nematodes (EPNs) that in some cases produce a nurse cell through their interactions with the plant cell from which they feed. Like symbiosis, scientific descriptions have identified EPN-governed nurse cell formation occurring in all groups of land plants including bryophytes, ferns, gymnosperms, angiosperms, and even multicellular algae (Cobb, 1890, 1893, 1930; Barton, 1892; Dixon, 1908; Bird and DiGennaro, 2012). These observations indicate that a common and ancient circuitry is in place that regulates these processes, but unlike symbiosis, EPNs would co-opt them to facilitate parasitism. Several studies show nodulation and other symbiotic processes to be affected by EPN infection, indicating the organisms affect the same metabolic processes (Barker et al., 1971, 1972; Lehman et al., 1971; Hussey and Barker, 1976; Huang and Barker, 1983; Ko et al., 1984; Pawlowski and Hartman, 2020). This outcome is not surprising since AM and nodulation involve inner cortical root cells occurring in the vicinity of where syncytia (pericycle and surrounding cells) are produced by syncytium-forming EPNs like *Heterodera glycines* and vascular cells interacting with giant-cell producing EPNs such as *Meloidogyne* sp.

Very little functional information exists on the genetic program that underlies the compatibility of plants to EPNs even though many plants are susceptible to their infection. In this regard, *G. max* has become an important model for studying plant-EPN activities because it can undergo compatible and incompatible interactions with both giant cell and syncytium-forming EPNs while still being able to undergo interactions with symbiotic organisms (Rebois et al., 1970; Kirkpatrick and May, 1989; Opperman and Bird, 1998; Pueppke et al., 1998; Machado and Krishnan, 2003; Niblack et al., 2006; Matsye et al., 2011, 2012; Cook et al., 2012; Liu et al., 2012). EPN-induced nurse cell formation, leading to the production of a syncytium or giant cell-containing galls, involves reprogramming the metabolic processes of those specific root cells to provide the EPN with its nutritional needs (Balasubramanian and Rangaswami, 1962; Chitwood and Lusby, 1991). The plant cell can interfere with the deployment and engagement of these injected materials by apparently transducing signals in some manner as an effective defense response (Ross, 1958; Endo and Veech, 1970; Gipson et al., 1971; Riggs et al., 1973; Endo, 1991). Such expressed defense genes in *G. max* include alpha soluble NSF attachment protein (α-SNAP), present in the *resistance to heterodera glycines 1* (*rhg1*) locus, and serine hydroxymethyltransferase (*Rhg4*) (Matsye et al., 2011, 2012; Cook et al., 2012; Liu et al., 2012; Matthews et al., 2013). Meanwhile, plants preserve their ability to engage in symbiosis if they are genetically capable of doing so (Kennedy et al., 1999).

Plant defense occurs through its perception of the pathogen. One defense signaling branch involves plant pathogen recognition receptors (PRRs) that recognize pathogen associated molecular patterns (PAMPs) to effect PAMP triggered immunity (PTI) (Jones and Dangl, 2006). Part of this response involves the release of Ca^2+^ into the cytoplasm (Ranf et al., 2014). A second branch, involving the perception of pathogen effectors, leads to effector triggered immunity (ETI) which if strong enough results in a hypersensitive response (HR) that leads to the sacrifice (death) of the affected cells/tissues (Jones and Dangl, 2006). PTI and ETI receptors signal through MAPKs leading to defense to *H. glycines* (Gopalan et al., 1996; Desikan et al., 1998; Asai et al., 2002; Day et al., 2006; Chinchilla et al., 2007; Aljaafri et al., 2017; McNeece et al., 2017, 2019; Klink et al., 2021a). MAPK expression leads to increased relative transcript abundances (RTAs) of genes within the *H. glycines*-induced syncytia undergoing a defense response that also have a demonstrated function in the defense response (Matsye et al., 2012; Pant et al., 2014; Sharma et al., 2016, 2020; Klink et al., 2017, 2021a; McNeece et al., 2017, 2019). Consequently, the identification of CSP gene expression occurring in syncytia undergoing the defense response indicates they may have a dual function in symbiosis and defense (Klink et al., 2009, 2010a,b, 2011, 2021a,b). Importantly, symbiosis and defense recruit calcium signaling with plant defense processes recruiting Ca^2+^ signaling in the cytoplasm while symbiosis employs nuclear Ca^2+^ signaling (Lévy et al., 2004; Mitra et al., 2004; Kwaaitaal et al., 2011; Ranf et al., 2011, 2014; Maintz et al., 2014; Keinath et al., 2015). Consequently, commonalities between the two processes likely involve Ca^2+^ at some level.

In the analysis presented here, *G. max DMI1, DMI2*, and *DMI3* genes are shown to be expressed during its defense response to *H. glycines* in parasitized root cells undergoing a defense response. Several paralogs compose each of the *G. max DMI1, DMI2*, and *DMI3* gene families. Through transgenic analyses, the *DMI1-3, DMI2-7*, and *DMI3-2* paralogs expressed within the *H. glycines*-parasitized syncytia undergoing the defense response are shown to function in the defense process. RNA sequencing (RNA-seq) analyses of RNA isolated from roots overexpressing defense *MAPK*s also exhibit increased RTAs of some *DMI* paralogs (McNeece et al., 2019). Furthermore, transgenic roots overexpressing *DMI1-3, DMI2-7*, and *DMI3-2* in some cases exhibit increased *MAPK3* RTAs. In contrast, transgenic roots undergoing RNAi of *DMI1-3, DMI2-7*, and *DMI3-2* in some cases exhibit decreased *MAPK3* RTAs. The combined results indicate *DMI* gene expression correlates with their ability to function in the defense response to *H. glycines* parasitism. *MAPK*s which function in the defense response are shown to regulate the expression of some of these *DMI* genes. Lastly, *DMI* genes are shown to regulate the expression of *MAPK3* which functions in the defense response possibly indicating the *DMI3-2* and *MAPK3-1* genes function in a co-regulated signal transduction loop.

## Materials and Methods

### Selection of Candidate Genes

Laser microdissection (LM) of *H. glycines*-induced feeding structures (syncytia) developing from parasitized root cells (pericycle) undergoing the process of defense is part of the experimental process used to identify the *DMI* genes under study (Klink et al., 2005, 2010a, 2021a). Two different *H. glycines*-resistant *G. max* genotypes are experimented on to identify consistently expressed candidate defense genes (Klink et al., 2011, 2021a; Matsye et al., 2011). To identify the *DMI* genes, *H. glycines*-resistant *G*.*max*_[Peking/PI548402]_ and *G*.*max*_[PI88788]_ are infected with *H*.*glycines*_[NL1−Rhg/HG−type7/race3]_, generating a defense response (Klink et al., 2021a). Roots are processed for paraffin-embedding and histology, followed by LM (Klink et al., 2005, 2007, 2009, 2010a,b, 2011, 2017, 2021a,b). RNA is isolated from LM-collected control cells (pericycle) sampled at 0-days post infection (dpi), prior to infection, and syncytia undergoing the process of defense at 3- and 6 dpi. The 3-dpi time point occurs prior to the onset of visible (histological) signs of a defense response while by 6-dpi the defense response is clearly different than a susceptible reaction (Ross, 1958; Endo, 1965, 1991; Pant et al., 2014). RNA is isolated using the PicoPure RNA Isolation kit (Molecular Devices®) with a DNAse treatment added just before the second column wash using DNAfree® (Ambion®). RNA yield and quality are determined using the RNA 6000 Pico Assay® (Agilent Technologies®) using the Agilent 2100 Bioanalyzer® according to manufacturer protocol. The cDNA probe preparation and hybridization on the Affymetrix® Soybean GeneChip® are performed according to Affymetrix® guidelines (Affymetrix®), run in triplicate for each of the *G*.*max*_[Peking/PI548402]_ and *G*.*max*_[PI88788]_ genotypes (Klink et al., 2007, 2009, 2010a,b, 2011; Matsye et al., 2011). Genes are considered expressed at a particular time point in the detection call methodology (DCM) if probe signal is measurable above threshold on all three arrays for that time point for both *G*.*max*_[Peking/PI548402]_ and *G*.*max*_[PI88788]_ (6 total arrays), *p* < 0.05 using the Bioconductor implementation of the standard Affymetrix® DCM (Klink et al., 2010a). The standard Affymetrix® microarray DCM analysis done in Bioconductor consists of four steps including (1) saturated probe removal, (2) discrimination score calculation, (3) Wilcoxon's rank test *p*-value calculation, and (4) detection call assignment. The quantitative procedure determines if the gene's expression is provably different from zero (present [P]), has uncertain measurement (marginal [G]), or is not provably different from zero (absent [A]). In the analysis presented here, a *DMI* gene meets the measured [M] criteria when the probe signal is detectable above threshold (*p* < 0.05) on all 6 arrays for a given time point. In contrast, the expression of a *DMI* gene is considered not measured (NM) if probe signal is not detected at a statistically significant level (*p* ≥ 0.05) on any one of the 6 arrays using the Mann–Whitney–Wilcoxon (MWW) Rank-Sum Test (Mann and Whitney, 1947). The MWW Rank Sum Test is a non-parametric test of the null hypothesis not requiring the assumption of normal distributions (Mann and Whitney, 1947). Some genes have no probe set fabricated onto the microarray. Consequently, gene expression is not determined and is not applicable (n/a). Gene accession numbers are provided from 2 different *G. max* genome annotations. For the microarray analysis, the Affymetrix annotations are mapped to the original *G. max* genome release (Accession 1) Wm82.a1.v1.1 (2010). This annotation had to be used at that time of publication of the work of Matsye et al. (2011) because just that annotation had been available. These older annotations have undergone a comparison here to update the accessions to the more recent *G. max* Wm82.a2.v1 (2015) genome assembly and annotation (Accession 2).

### *G. max DMI* Gene Identification

All of the *G. max* DMI1, DMI2, and DMI3 protein family sequences are identified at Phytozome (https://phytozome.jgi.doe.gov) using the 881 amino acid (aa) *M. truncatula* DMI1 (AAS49490), the 925 aa DMI2 (Q8L4H4), and the 523 aa DMI3 (Q6RET7) protein sequences (Schmutz et al., 2010; Goodstein et al., 2012). The processes involved in the *G. max* proteome query include the use of the Basic Local Alignment Search Tool program (BLAST) (Altschul et al., 1990). The parameters of the BLAST query are the default settings, including Target type: Proteome; Program: BLASTP-protein query to protein database; Expect (E) threshold:−1; Comparison matrix: BLOSUM62; Word (W) length: default = 3; number of alignments to show: 100 allowing for gaps and filter query, in order that they appear on the BLAST program.

### PCR Primer Design

The *G. max DMI1, DMI2*, and *DMI3* cDNA sequences are acquired from Phytozome (Schmutz et al., 2010; Goodstein et al., 2012). DNA primer sequences are designed for OE of the full length targeted *DMI* genes in the pRAP15-*ccd*B destination vector and RNAi in the pRAP17-*ccd*B RNAi destination vector (Supplementary Table 1) (Klink et al., 2009, 2021a; Matsye et al., 2012). The nucleotide sequence, CACC, is added to the 5' end of the forward PCR primer for directional cloning into the pENTR™ entry vector (Invitrogen). The control used for the RT-qPCR analyses is the *G. max* ribosomal protein gene *RPS21* (Glyma.15G147700), proven to be transcribed into mRNA and translated into protein (Morita-Yamamuro et al., 2004; Klink et al., 2005; Matsye et al., 2012).

### *DMI* Gene Cloning

The RNeasy Plus Mini Kit and protocol (Qiagen®) are used to isolate mRNA. SuperScript First Strand Synthesis System for RT-PCR (Invitrogen®) with oligo d(T) as the primer are used with their protocol to make cDNA template for PCR cloning using the appropriate primers (Supplementary Table 1). Using designed PCR primers, genes are PCR-amplified from the cDNA template (Niraula et al., 2020). PCR amplification of targeted *DMI* genes occurs with high fidelity Platinum® taq (Invitrogen®) according to their protocol. PCR conditions include DNA dissociation for 10 min at 96°C with subsequent PCR cycling and temperature set for denaturation for 30 s at 96°C, annealing for 60 s at 55°C and extension for 30 s at 72°C for 35 cycles, terminating at 4°C. PCR reactions, separated by gel electrophoresis, are run on a 1% TAE agarose gel. *DMI* gene products (amplicons) corresponding to correct size are excised from the gel with a fresh, unused, sterile razor blade. The *DMI* DNA amplicons are isolated from the agarose gel using the X-TRACTA gel extractor (USA Scientific) and purified using the QIAquick Gel Extraction Kit (Qiagen®) according to their protocol. The purified *DMI* DNA is used for Gateway® cloning (Karimi et al., 2002, 2007; Curtis and Grossniklaus, 2003). The purified, PCR-generated, *DMI* amplicons are directionally cloned into the pENTR™ entry vector following the pENTR™/D-TOPO® protocol (Invitrogen). Transformation of the entry vector containing the *DMI* amplicon into One Shot® chemically competent *E. coli* cells is immediately followed by selection on LB-kanamycin (LB-kan) plates, 50 μg/ml (Invitrogen). Subsequently, the selected colony is transferred to LB-kan broth, 50 μg/ml, followed by incubation for 12–14 h in a 37°C shaker at 225 rpm. Plasmid DNA is isolated from selected colonies using the Wizard *Plus* SV Minipreps DNA Purification System (Promega) according to the manufacturer's instructions. The *DMI* genes are engineered into the pRAP15-*ccd*B destination vector for OE of the gene and the pRAP17-*ccd*B destination vector for RNAi of the gene following the LR Clonase II kit and protocol (Invitrogen). The LR clonase reaction replaces the *ccd*B gene with the *DMI* amplicon. The pRAP15-*ccd*B and pRAP17-*ccd*B vectors have the figwort mosaic virus (FMV) sub-genomic transcript (Sgt) promoter to drive target (*DMI*) gene expression (Klink et al., 2009, 2021a; Matsye et al., 2012). The FMV-Sgt sequence is a 301-bp FMV-Sgt promoter fragment (sequence −270 to +31 from the transcription start site [TSS]) (Bhattacharyya et al., 2002). The pRAP15 and pRAP17 plasmids contain an enhanced green fluorescent protein (eGFP) gene driven by the *rol*D promoter and which is terminated by t35S translational terminator for effective visual reporting in plant tissue (White et al., 1985; Elmayan and Tepfer, 1995; Haseloff et al., 1997; Klink et al., 2021a). The reaction contents containing the pRAP15 and pRAP17 destination vectors undergoing the LR reaction to ligate the *DMI* gene amplicons are then transformed using One Shot® chemically competent *E. coli* cells and protocol (Invitrogen). Colony selection is performed on LB-tet plates, 5 μg/ml. Transformed One Shot® *E. coli* bacteria having the plasmid DNA are used to inoculate LB-tet broth, 5 μg/ml, incubated for 12–14 h in a 37°C shaker at 225 rpm. The plasmid DNA is isolated as described and confirmed for the presence of the *DMI* gene by PCR using the appropriate PCR primers. The *DMI* gene-containing destination vector DNA is used to transform *Agrobacterium rhizogenes* (K599) using the freeze thaw method (Hofgen and Willmitzer, 1988). Colonies are selected on LB-tet plates, 5 μg/ml. Positive colonies are tested for the presence of eGFP, root inducing (Ri) plasmid, and the *DMI* gene by PCR using the appropriate primers (Ryder et al., 1985; Haseloff et al., 1997; Hodges et al., 2004; Pant et al., 2016; McNeece et al., 2019) (Supplementary Table 1). Details of the pRAP15 and pRAP17 plasmids are available (Klink et al., 2021a).

### *G. max* Genetic Transformations

Transgenic OE and RNAi for the respective *DMI* genes began when the *G. max* root of a 1-week-old plant is removed at the hypocotyl with a new, sterile razor blade (Pant et al., 2014). The *H. glycines*-susceptible, *resistant to Heterodera glycines1* (*rhg1*)-lacking (*-/-*), (*rhg*1^−/−^), *G*.*max*_[Williams82/PI518671]_ genetic background is used in the OE experiments of *DMI1-3, DMI2-7*, and *DMI3-2* (Bernard and Cremeens, 1988; Atkinson and Harris, 1989; Schmutz et al., 2010). The *H. glycines*-resistant *rhg1*-containing (+/+), (*rhg*1^+/+^), *G*.*max*_[Peking/PI548402]_ genetic background is used for RNAi studies of *DMI1-3, DMI2-7*, and *DMI3-2* (Ross, 1958). To control for non-specific effects of target gene expression, controls for each experiment are constructed by transforming the respective genotype with pRAP15 or pRAP17 vector having the *ccd*B gene in place of the *DMI* gene sequence (pRAP15-*ccd*B or pRAP17-*ccd*B) (Pant et al., 2014). The pRAP15-*ccd*B OE control is produced in *G*.*max*_[Williams82/PI518671]_. The pRAP17-*ccd*B RNAi control is produced in *G*.*max*_[Peking/PI548402]_. The hypocotyl is immersed in the transformed K599 cell solution in Murashige and Skoog (MS) medium in suspension in a Petri dish with the root then being removed to permit the transformed K599 cells to gain access to the plant tissue (Murashige and Skoog, 1962). A group of 25 root-less plants is placed in a 140-ml glass beaker containing 25 ml of transformed K599 cells in MS medium in suspension. The plants are placed under vacuum using the VP60 Two Stage Vacuum Pump (CPS Products, Inc.) in a Bel-Art Space Saver polycarbonate vacuum desiccator with a clear polycarbonate bottom for 5 min and then left under vacuum for 10 min. The vacuum is then slowly released to allow the transformed K599 cells to further enter the plant tissue. After this cocultivation period, the cut ends of the root-less plants are individually placed 3–4 cm deep into fresh coarse grade A-3 vermiculite (Palmetto Vermiculite). The vermiculite is placed in and then pre-wetted with distilled water in 50-cell propagation trays (725602C) held in standard fats (710245C) with holes in the bottom (T.O. Plastics). The plant trays are placed in Sterlite® 25-qt./23-L modular latched boxes then covered with their lids. The covered modular latched boxes are placed 20 cm from standard fluorescent cool white 4,100-K/32-W bulbs emitting 2,800 lumens (Sylvania). The boxes remain under the lights for 5 days at ambient laboratory temperature (22°C). The plants are subsequently transferred to the greenhouse where the plants in the trays are removed from the modular latched boxes. The plants recover in the greenhouse for 1 week. Visual selection of transgenic *G. max* roots is carried out using the eGFP reporter, employing a Dark Reader Spot Lamp (SL10S) (Clare Chemical Research) (Klink et al., 2021a). Roots exhibiting eGFP reporter expression also possess the *DMI* gene expression cassette, each having their own promoter and terminator sequences (Klink et al., 2021a). Gene transfer happens because the K599 cells have the capability to facilitate the transport of the DNA cassettes present between the left and right borders of the pRAP15 and pRAP17 destination vectors into the somatic root cell chromosomal DNA. Even though the DNA cassette is not incorporated into the germline, the result is a stable transformation event occurring in the root somatic cell. Roots subsequently develop from the transgenic cell over a period of a few weeks. The resultant genetically mosaic plants have a non-transgenic shoot with a transgenic root system. Therefore, each individual transgenic root system is an independent transformant line. The transgenic plants are each planted in a Ray Leach Conetainer (SC10) (Stuewe and Sons, Inc.) having a cell diameter of 3.81 cm (1.5 in), a depth of 20.96 cm (8.25 in), and a volume of 164 ml in sandy (93.00% sand, 5.75% silt, and 1.25% clay) soil and allowed to recover for 2 weeks prior to the start of the experiment. The conetainers are secured in a Ray Leach Tray (RL98) (Stuewe and Sons, Inc.). The functionality of the genetic constructs (i.e., RTA increased in OE roots and RTA decreased in RNAi roots) in *G. max* is confirmed by real-time quantitative PCR [RT-qPCR].

### Real-Time Quantitative PCR

Gene-specific RT-qPCR primers are designed for the *DMI1-3, DMI2-7*, and *DMI3-2* gene family members, to carry out the RTA analyses (Supplementary Table 1). The cDNA prepared in the experiments is constructed from mRNA collected from the transgenic roots at 0-dpi, prior to infection but mock-inoculated, and used to confirm the expression of the respective targeted OE or RNAi of the *DMI* transgenes as described. The RTA of the candidate defense genes in the transgenic roots is confirmed using the already-described *G. max RPS21*. Analyses of the non-targeted *DMI* genes, and remaining genes in the *G. max* genome is beyond the scope of the confirmation process. The RT-qPCR experiments utilize the Taqman 6 carboxyfluorescein (6-FAM) probes with the Black Hole Quencher (BHQ1) (MWG Operon; Birmingham, AL). The qPCR reaction is accomplished by preincubation at 50°C for 2 min, followed by 95°C for 10 min. Proceeding from this step is alternating 95°C for 15 s then 60°C for 1 min for 40 cycles (Matsye et al., 2012). The statistical analysis using 2^−Δ*ΔCT*^ to calculate fold change is followed according to the derived formula presented in Livak and Schmittgen (2001). The results have been tested statistically using the Student's *t*-test (*p* < 0.05) (Yuan et al., 2006).

### The Infection of *G. max* by *H. glycines*, Cyst Extraction, Female Index Calculation and Root Mass Determination

The *H*.*glycines*_[NL1−Rhg/HG−type7/race3]_ is used for the infection of the transgenic roots. This choice is made because of its effectiveness in parasitizing *G*.*max*_[Williams82/PI518671]_ and failure to successfully parasitize *G*.*max*_[Peking/PI548402]._ *H. glycines* females are isolated by sucrose flotation (Jenkins, 1964; Matsye et al., 2012). The standard *H. glycines*-susceptible *G*.*max*_[Williams82/PI518671]_ is used for experiments requiring a susceptible genotype. The *H. glycines*-resistant *G*.*max*_[Peking/PI548402]_ is used for experiments requiring a resistant genotype. The second stage juveniles (J2s) are hatched and concentrated to a final inoculum concentration of 2,000 J2s/ml (Matsye et al., 2012). The inoculum (1 ml) is dispensed into 7 mm diameter holes made near the base of the plant. This procedure directs the J2s to the root system. Once the inoculum is dispensed and absorbed into the soil, the holes are covered to prevent expulsion of the nematodes by subsequent watering. After 30 days, test roots are stained with acid fuschin to confirm infection (Byrd et al., 1983). The remaining roots from that replicate experiment are then processed for extraction of cysts from the soil to calculate the female index (FI) (Golden et al., 1970). *H. glycines* cyst extraction involves taking each individual plant and massaging the transgenic root to release the cysts from the soil/root system (Klink et al., 2009). The soil containing the cysts is repeatedly washed and the rinsed water filtered over a 20-mesh sieve nested within a 100-mesh sieve (Matsye et al., 2012). The outcome is the collection of all cysts (Matsye et al., 2012).

*H. glycines* cyst count and root mass are enumerated for each plant and used to calculate the female index (FI) as it relates to the whole root (wr) system and cysts per gram (pg) of root system (McNeece et al., 2019). The FI is the community standard, acknowledged evaluation for interpreting the effects of a condition on *H. glycines* (Golden et al., 1970). The approach is used in order to standardize the enumeration of cysts for the FI calculation. The wr analysis procedure is the historically performed method used to enumerate cysts which does not consider the effect the plant genotype or transgenic event has on *H. glycines* parasitism (McNeece et al., 2019). The pg analysis procedure is employed in order to consider the effect the plant genotype or transgenic event has on *H. glycines* parasitism since the calculation of the FI adjusts for root mass (McNeece et al., 2019).

The FI is calculated as FI = (Nx/Ns) X 100 (Golden et al., 1970). Nx is the average number of females on the test cultivar (Golden et al., 1970). Ns is the average number of females on the standard susceptible cultivar (Golden et al., 1970). Nx in the experiments presented here accounts for the pRAP15 containing the *DMI* where it is being overexpressed (*DMI*-OE) for its targeted increase in RTA or pRAP17 containing the *DMI* RNAi (*DMI*-RNAi) where the gene is targeted to decrease its RTA. Ns accounts for the engineered OE control containing the pRAP15-*ccd*B or RNAi control containing the pRAP17-*ccd*B non-engineered, empty vectors described previously. The pRAP15 and pRAP17 plasmids are not empty *per se* as they have the *ccd*B gene that functions as a control. The wr and pg FI calculations are tested statistically using the Mann–Whitney–Wilcoxon (MWW) Rank-Sum Test, *p* < 0.05 (Mann and Whitney, 1947; Matsye et al., 2012). The study incorporates three biological replicates, with at least 10 individual experimental replicates in each biological replicate. In the study presented here, the number of analyzed transgenic roots for *DMI1-3*-OE (*n* = 30), *DMI2-7*-OE (*n* = 30), and *DMI3-2* -OE (*n* = 30), (10 roots per replicate) are compared to the pRAP15-*ccd*B control (*n* = 36) (at least 10 roots per replicate). The number of transgenic roots analyzed for *DMI1-3*-RNAi (*n* = 30), *DMI2-7*-RNAi (*n* = 30), and *DMI3-2*-RNAi (*n* = 30), (10 roots per replicate) are compared to the pRAP17-*ccd*B control (*n* = 34), having at least 10 roots per replicate.

### RNA-Seq Gene Expression Analyses of Transgenic Root RNA

Prior functional transgenic analyses of the 32-member *G. max* MAPK gene family determine that 9 of them function in the defense response to *H. glycines* parasitism and are referred to as defense MAPKs (McNeece et al., 2019). The defense MAPKs undergoing overexpression (OE) or RNA interference (RNAi) include *MAPK2* (Glyma.06G029700), *MAPK3-1* (Glyma.U021800), *MAPK 3-2* (Glyma.12G073000), *MAPK 4-1* (Glyma.07G066800), *MAPK 5-3* (Glyma.08G017400), *MAPK6-2* (Glyma.07G206200), *MAPK 13-1* (Glyma.12G073700), *MAPK16-4* (Glyma.07G255400), and *MAPK20-2* (Glyma.14G028100) with OE and RNAi root samples collected from each for RNA-seq. The pRAP15-*ccd*B and pRAP17-*ccd*B control root samples are collected for RNA-seq (Alshehri et al., 2019). RNA is isolated from the collected root samples as already described. The collected samples are validated, sequenced, and analyzed, producing Illumina® RNA-seq data for use in examining gene expression of the 55,022 genes in the *G. max* genome (Alshehri et al., 2019). The RNA-seq fold change (FC) data, representing the relative transcript abundance (RTA) is mined specifically for *DMI* gene paralog expression (Wang and Wang, 2021). The FC for the OE experiments is determined in comparisons of the transgenic *MAPK-*OE RNA-seq data as compared to the RNA-seq data obtained from the transgenic pRAP15-*ccdB* (overexpression) control (Wang and Wang, 2021). The FC for the RNAi experiments is determined in comparisons of the transgenic *MAPK-*RNAi RNA-seq data as compared to the RNA-seq data obtained from the transgenic pRAP17*-ccdB* (RNAi) control (Wang and Wang, 2021). When presented, confirmation of the RNA-seq RTAs, given as FC, is performed by RT-qPCR as described (Livak and Schmittgen, 2001; Klink et al., 2021a).

### Proteome Mining

DMI homologs and splice variants from various agricultural crops of international importance and select importance in the U.S. are identified (Tilman et al., 2011; Ray et al., 2013, 2019; Burkhead and Klink, 2018). Analyses are performed by BLASTing selected conceptually translated genes to the described protein coding regions of genomes with the *M. truncatula* DMI1, DMI2, and DMI3 protein sequences. The proteomes, in addition to *G. max* (G.max Wm82.a2.v1), include *Manihot esculenta* (M.esculenta v8.1), *Zea mays* (Z.mays RefGen_V4)*, Oryza sativa* (O.sativa v7.0), *Triticum aestivum* (T.aestivum v2.2), *Hordeum vulgare* (H.vulgare r1), *Sorghum bicolor* (S.bicolor v3.1.1)*, Brassica rapa* (B.rapaFPsc v1.3), *Solanum tuberosum* (S.tuberosum v6.1), *S. lycopersicum* (S.lycopersicum ITAG4.0), *Gossypium hirsutum* (G.hirsutum v2.1), and *B. vulgaris* (B.vulgaris EL10_1.0) which are housed at Phytozome under default settings (Goodstein et al., 2012).

## Results

### The Identification of *DMI* Gene Families and Root Cell-Specific Expression

An analysis was performed that compared the gene expression which occurred in control (pericycle) cells (0 dpi), and within *H. glycines*-induced syncytia at 3, and 6 dpi in 2 different genotypes (*G*.*max*_[Peking/PI548402]_ and *G*.*max*_[PI88788]_), each capable of mounting a defense response. The two different *H. glycines*-resistant *G. max* genotypes were experimented on to identify consistently expressed candidate defense genes (Klink et al., 2011, 2021a; Matsye et al., 2011). The results from those experiments led to the identification of an annotated *G. max* gene homologous to the *M. truncatula DMI3*. *DMI3* did not exhibit expression at the 0 dpi time point but exhibited expression in syncytia that has undergone the defense response in *G*.*max*_[Peking/PI548402]_ and *G*.*max*_[PI88788]_ at 3, and 6 dpi (Table 1 and Supplementary Table 2). The CSP has 3 different *DMI* genes, *DMI1, DMI2*, and *DMI3*. BLAST analyses of the *G. max* proteome identified 3 DMI1, 12 DMI2, and an additional DMI3 paralog (Supplementary Tables 2, 3). Further examination of previously generated transcriptomic data identified the expression activity for the *G. max DMI1, DMI2*, and *DMI3* paralogs that occurred while a defense response was mounted to *H. glycines* parasitism (Table 1 and Supplementary Tables 2, 3). The experiment demonstrated at least one paralog for each *DMI* gene family gene underwent expression during the defense response.

**Table 1:** *G. max DMI* genes identified as being expressed in syncytia that were undergoing a defense response to *H. glycines* parasitism.

**Gene**	**0**	**3**	**6**
**Time point**			
*DMI1-3*	NM	M	M
*DMI2-7*	NM	NM	M
*DMI3-2*	NM	NM	M

*The conceptually translated DMI genes were used to identify their protein family paralogs from the G. max proteome according to the procedures outlined in the Materials and Methods subsection: G. max DMI gene identification. The DCM data for the DMI genes were obtained according to the procedures outlined in the Materials and Methods subsection: Selection of candidate genes. Terminology: Time points, the time (dpi) of H. glycines infection (0, 3, and 6 dpi) at which the G. max root cells were collected by laser microdissection (LM), and their isolated RNA used for the gene expression studies. Expression, not measured (NM) to statistically significant probability (p) value (p-value) levels (p ≥ 0.05); measured (M) to statistically significant levels (p < 0.05). Expression significance was calculated using the Mann–Whitney–Wilcoxon (MWW) Rank-Sum Test, significance at p < 0.05 (Mann and Whitney, 1947). Accompanying data has been presented in Supplementary Tables 2, 3*.

BLAST queries of globally important crop proteomes, and some with more importance to U.S. production, identified DMI1, DMI2, and DMI3 homologs, and in some cases, additional paralogs (Table 2 and Supplementary Tables 4–6). Genomic data on alternative splice variants were also identified, provided here since alternate splice variants of other *G. max* genes function in the defense response that *G. max* has to *H. glycines* (Supplementary Tables 4–6). The *DMI1-3* (Glyma.19G263500), *DMI2-7* (Glyma.11G246200), and *DMI3-2* (Glyma.15G222300) paralogs that exhibited expression during the defense response in syncytia were selected for functional transgenic experiments as the approach serves as an effective strategy in identifying defense genes.

**Table 2:** DMI paralogs present in select crop species.

**Species**	**DMI1**	**DMI2**	**DMI3**
*G. max*	3	2 (11)	2
*G. hirsutum*	4	2 (6)	2
*M. esculenta*	2	1 (9)	1
*Z. mays*	2	2	1 (2)
*O. sativa*	2	1 (5)	1
*T. aestivum*	7 (8)	3 (7)	3
*H. vulgare*	2	1 (5)	2
*S. bicolor*	2	1 (8)	1
*B. rapa*	1	0 (16)	0
*B. vulgaris*	1	0 (3)	0
*S. lycopersicon*	1	1 (4)	1
*S. tuberosum*	2	1 (4)	1

*Values not in parentheses had a Blast of e-0. Numbers in parentheses had a Blast cutoff of > e-0. Please refer to Materials and Methods subsections: G. max DMI gene identification, and Proteome mining, for details. Raw Blast data has been provided (DMI1-Supplementary Table 4, DMI2-Supplementary Table 5, DMI3-Supplementary Table 6)*.

### *DMI* Relative Transcript Abundance Changes Occurred When Experimentally Targeted

**Figure 1:**
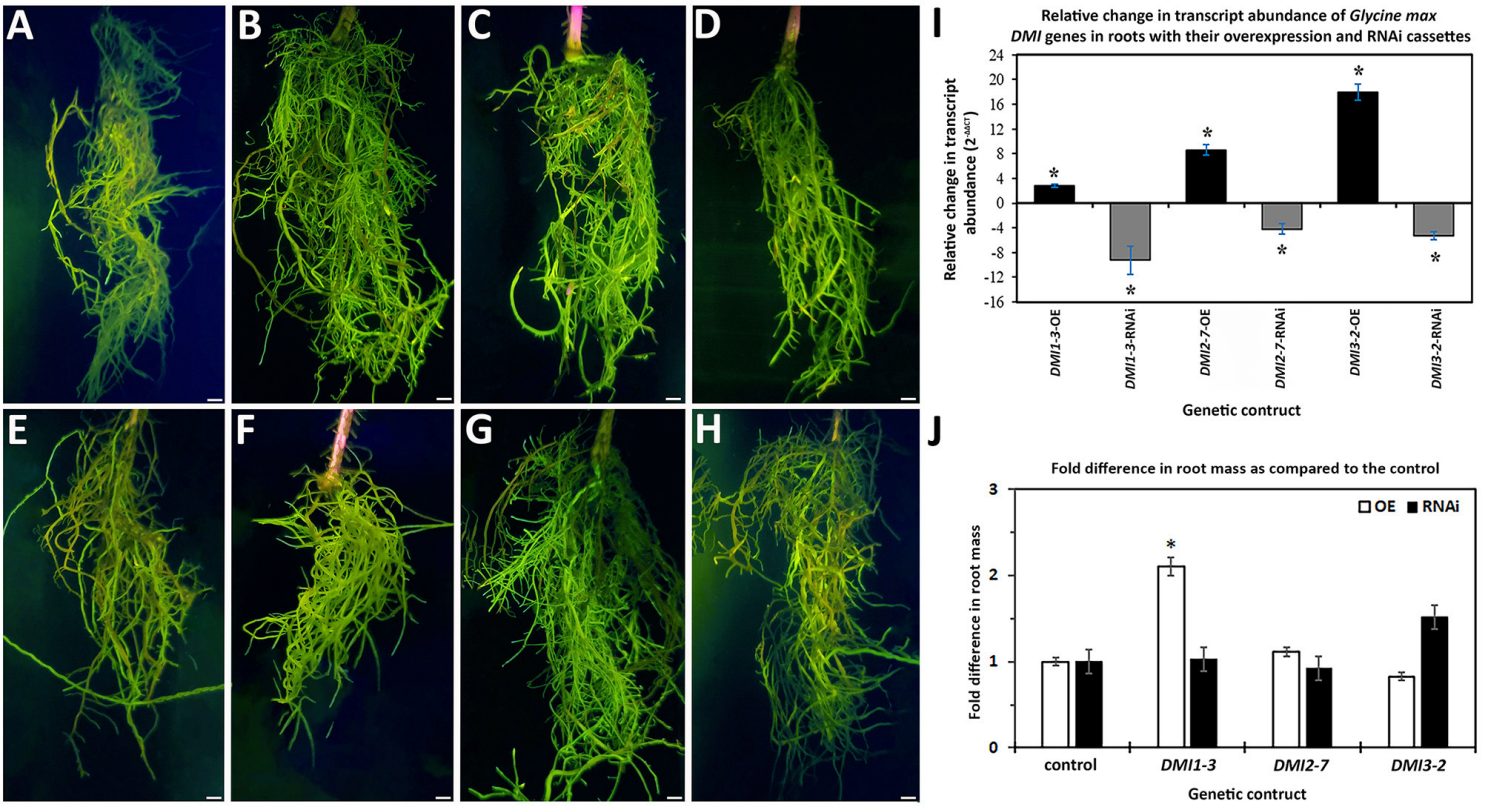
Transgenic roots obtained from chimeric plants having genetically engineered roots and un-engineered shoots that were generated through the K599-mediated genetic transformation procedure. Please refer to Materials and Methods subsections: *DMI* gene cloning, and *G. max* genetic transformations, for details. The presented values represent the numbers of plants used in the image generation, and those roots studied in the *H. glycines* infection analyses. Terminology: overexpression (OE); RNA interference (RNAi); relative transcript abundance (RTA); fold change (FC); probability (*p*) value (*p*-value;); female index (FI). **(A)** pRAP15-*ccd*B (OE control) (*n* = 36); **(B)** *DMI1-3-*OE (*n* = 30); **(C)** *DMI2-*7-OE (*n* = 30); **(D)** *DMI3-2* -OE (*n* = 30); **(E)** pRAP17-*ccd*B (RNAi control) (*n* = 34); **(F)** *DMI1-3-*RNAi (*n* = 30); **(G)** *DMI2-*7-RNAi (*n* = 30); **(H)** *DMI3-2*-RNAi (*n* = 30). Bars = 1 cm. **(I)** The expected change in *DMI1-3, DMI2-7*, and *DMI3-2* RTA, presented as FC, for the -OE and -RNAi roots, as compared to their pRAP15-*ccd*B and pRAP17-*ccd*B overexpression and RNAI controls, respectively, was calculated by 2^−Δ*ΔCT*^ to determine FC (Livak and Schmittgen, 2001; Klink et al., 2021a). The RTA of the candidate defense genes, presented as a FC, in the transgenic roots was compared using the *G. max* ribosomal protein gene *RPS21* (Glyma.15G147700). The RT-qPCR analyses examined 3 experimental replicates (individual root systems) of *DMI1-3, DMI2-7*, and *DMI3-2* -OE or -RNAi roots as compared to their pRAP15-*ccd*B, and pRAP17-*ccd*B controls, respectively, from each of the 3 biological replicates. Each experimental replicate was run in triplicate using the same RNA. (*), statistical significance of *p* < 0.05, Student's *t*-test (Yuan et al., 2006). Please refer to Materials and Methods subsection: Real-time quantitative PCR (RT-qPCR), for details. **(J)** The effect that the expression of the *DMI1-3, DMI2-7*, or *DMI3-2* -OE or -RNAi cassettes had on root mass as compared to their respective pRAP15-*ccd*B or pRAP17-*ccd*B controls. The statistical significance (*) for the change in root mass was determined using the MWW Rank-Sum Test, *p* < 0.05 (Mann and Whitney, 1947). Please refer to Materials and Methods subsection: The infection of *G. max* by *H. glycines*, cyst extraction, FI calculation, and root mass, for details.

The *DMI1-3, DMI2-7*, and *DMI3-2* genes were targeted for experimentally altering their RTA through transgenic manipulation, presented as a fold change (FC) in expression as compared to the appropriate control. The *DMI1-3, DMI2-7*, and *DMI3-2* genes were engineered for OE in the *H. glycines*-susceptible *G*.*max*_[Williams82/PI518671]_, based on the hypothesis that their increase in RTA would make the *H. glycines*-susceptible *G*.*max*_[Williams82/PI518671]_ resemble the observed *H. glycines* defense response that occurs in *G*.*max*_[Peking/PI548402]_. In contrast, *DMI1-3, DMI2-7*, and *DMI3-2* genes were engineered for RNAi in the *H. glycines*-resistant *G*.*max*_[Peking/PI548402]_, based on the hypothesis that their decrease in RTA would make the *H. glycines*-resistant *G*.*max*_[Peking/PI548402]_ resemble the observed *H. glycines* susceptibility that occurs in *G*.*max*_[Williams82/PI518671]_. The combination of the hypothesized increase in *DMI1-3, DMI2-7*, and *DMI3-2* RTA in *G*.*max*_[Williams82/PI518671]_ that would lead to more *H. glycines*-resistant roots, and decrease in *DMI1-3, DMI2-7*, and *DMI3-2* RTA in the *G*.*max*_[Peking/PI548402]_ that would lead to more *H. glycines*-susceptible roots would be evidence that the targeted gene functioned in the defense response. Transgenic *DMI1-3, DMI2-7*, and *DMI3-2* -OE roots, and their respective transgenic pRAP15-*ccd*B OE control roots, were made (Figure 1). Transgenic *DMI1-3, DMI2-7*, and *DMI3-2*-RNAi roots, and their respective transgenic pRAP17-*ccd*B RNAi control roots, had then also been made (Figure 1). The expected change in *DMI1-3, DMI2-7*, and *DMI3-2* RTA, presented as a FC in gene expression for the -OE and -RNAi roots as compared to their respective transgenic controls using the *G. max RPS21* were determined (Figure 1). The increase in RTA in the *DMI1-3, DMI2-7*, and *DMI3-2* -OE roots ranged from 3 to 18-fold while the change in RTA in the *DMI1-3, DMI2-7*, and *DMI3-2*-RNAi roots ranged from−4 to−9-fold as compared to their controls (Figure 1). *DMI1-3, DMI2-7*, and *DMI3-*2 RTAs have also been determined at 6 dpi *H. glycines* infection in cDNA generated from their respective RNAs isolated from their OE and RNAi transgenic roots as compared to their controls (Supplementary Table 1). An examination of the effect that *DMI1-3, DMI2-7*, and *DMI3-2* -OE or-RNAi had on the remaining *DMI* genes were not performed.

An examination of root mass from *DMI1-3, DMI2-7*, and *DMI3-2* -OE and -RNAi roots as compared to their respective pRAP15-*ccd*B OE and pRAP17-*ccd*B RNAi controls presented in Figure 1 were performed. The analyses identified *DMI1-3*-OE transgenic roots exhibited affected growth to a statistically significant level as compared to the respective transgenic pRAP15-*ccd*B OE control roots. In this case, the statistically significant change in root mass observed for *DMI1-3*-OE roots was an increase of 2.10 fold as compared to its control (Figure 1). The remaining analyses of the other *DMI*-OE and -RNAi roots did not lead to the identification of a statistically significant effect on root mass as compared to their appropriate controls.

### Altering *G. max DMI* RTA Changes the Capability of *H. glycines* to Parasitize Roots

**Figure 2:**
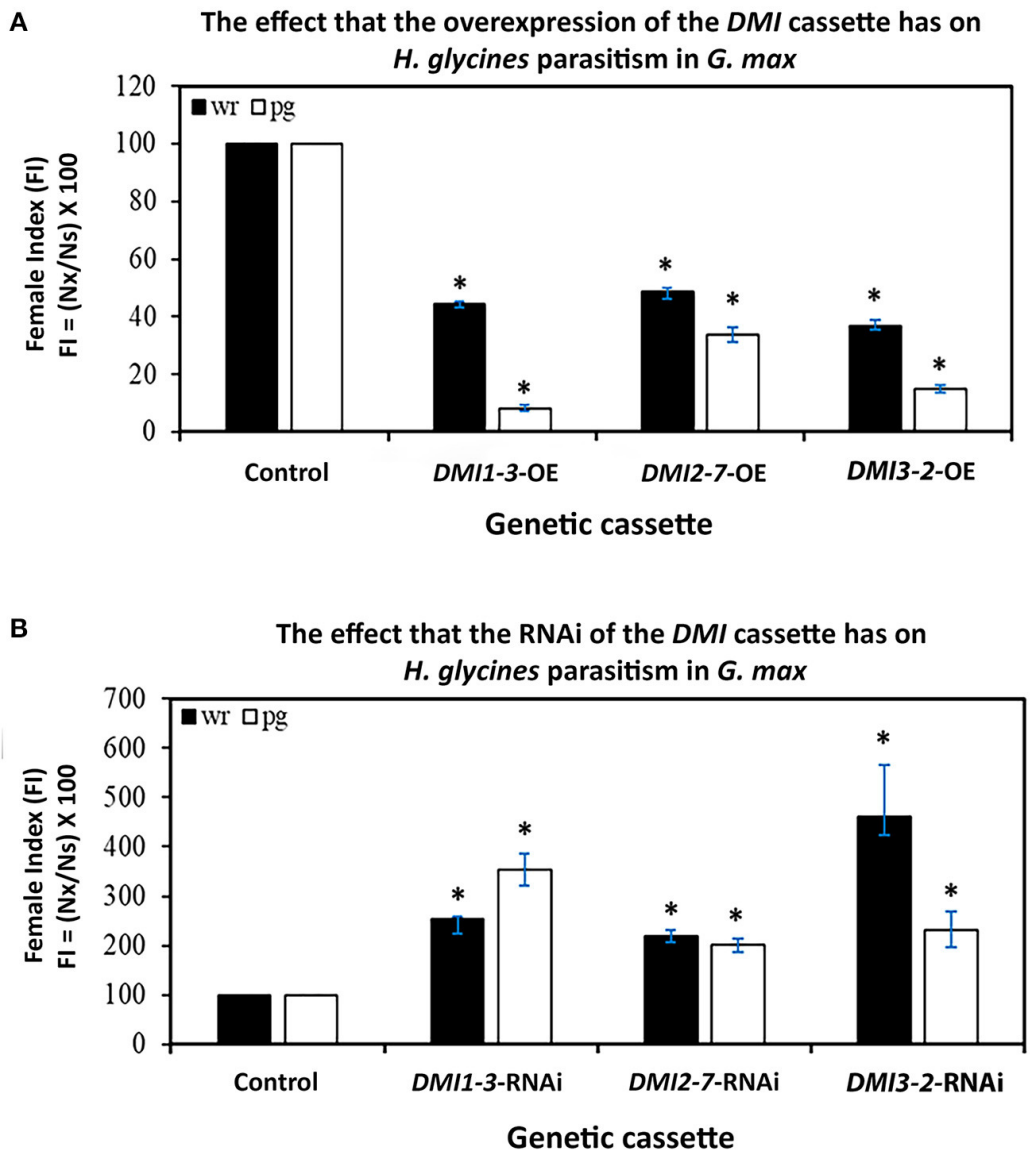
The FI for *DMI1-3, DMI2-7*, and *DMI3-2* -OE and -RNAi roots as compared to their respective pRAP15-*ccd*B and pRAP17-*ccd*B controls. The statistical significance (*) was determined by the MWW Rank-Sum Test, *p* < 0.05 (Mann and Whitney, 1947). The analyzed transgenic roots for *DMI1-3*-OE (*n* = 30), *DMI2-7*-OE (*n* = 30), and *DMI3-2*-OE (*n* = 30) were compared to the pRAP15-*ccd*B control (*n* = 36). The analyzed transgenic roots for *DMI1-3*-RNAi (*n* = 30), *DMI2-7*-RNAi (n = 30), and *DMI3-2*-RNAi (*n* = 30) were compared to the pRAP17-*ccd*B control (*n* = 34). Please refer to Materials and Methods subsection: The infection of *G. max* by *H. glycines*, cyst extraction and female index calculation, for details.

The FI studies of *H. glycines* parasitism in relation to the altered expression of the *DMI1-3* genes were performed, examining their pathogenic ability in relation to the enumerated cyst numbers in the wr and pg of root analyses. The analyses were done in this manner to gain insight as to whether the expression of the *DMI*-OE or *DMI*-RNAi gene cassettes affected root mass as compared to their controls and therefore skewed the FI analysis. The generation of *DMI1-3, DMI2-7, DMI3-2*-OE, and pRAP15-*ccd*B OE control roots occurred in the *H. glycines* susceptible *G*.*max*_[Williams 82/PI518671]_. In comparison to the generated pRAP15-*ccd*B OE control, the experimentally increased *DMI1-3, DMI2-7*, and *DMI3-2* RTA that occurred through their OE led to a statistically significant 52-63% suppression of *H. glycines* parasitism as compared to the control (Figure 2). In contrast, the generation of *DMI1-3, DMI2-7, DMI3-2*-RNAi, and pRAP17-*ccd*B RNAi control roots were made in the *H. glycines*-resistant *G*.*max*_[Peking/PI548402]_. In comparison to the pRAP17-*ccd*B RNAi controls, experimentally decreased *DMI1-3, DMI2-7*, and *DMI3-2* RTA that occurred by RNAi led to a statistically significant 2.1-to-4.6-fold increase in *H. glycines* parasitism as compared to te control (Figure 2). The combination of the two outcomes, opposite in nature, is taken as evidence that the gene functions during the defense response.

### MAPKs Influence *DMI* Expression

**Figure 3:**
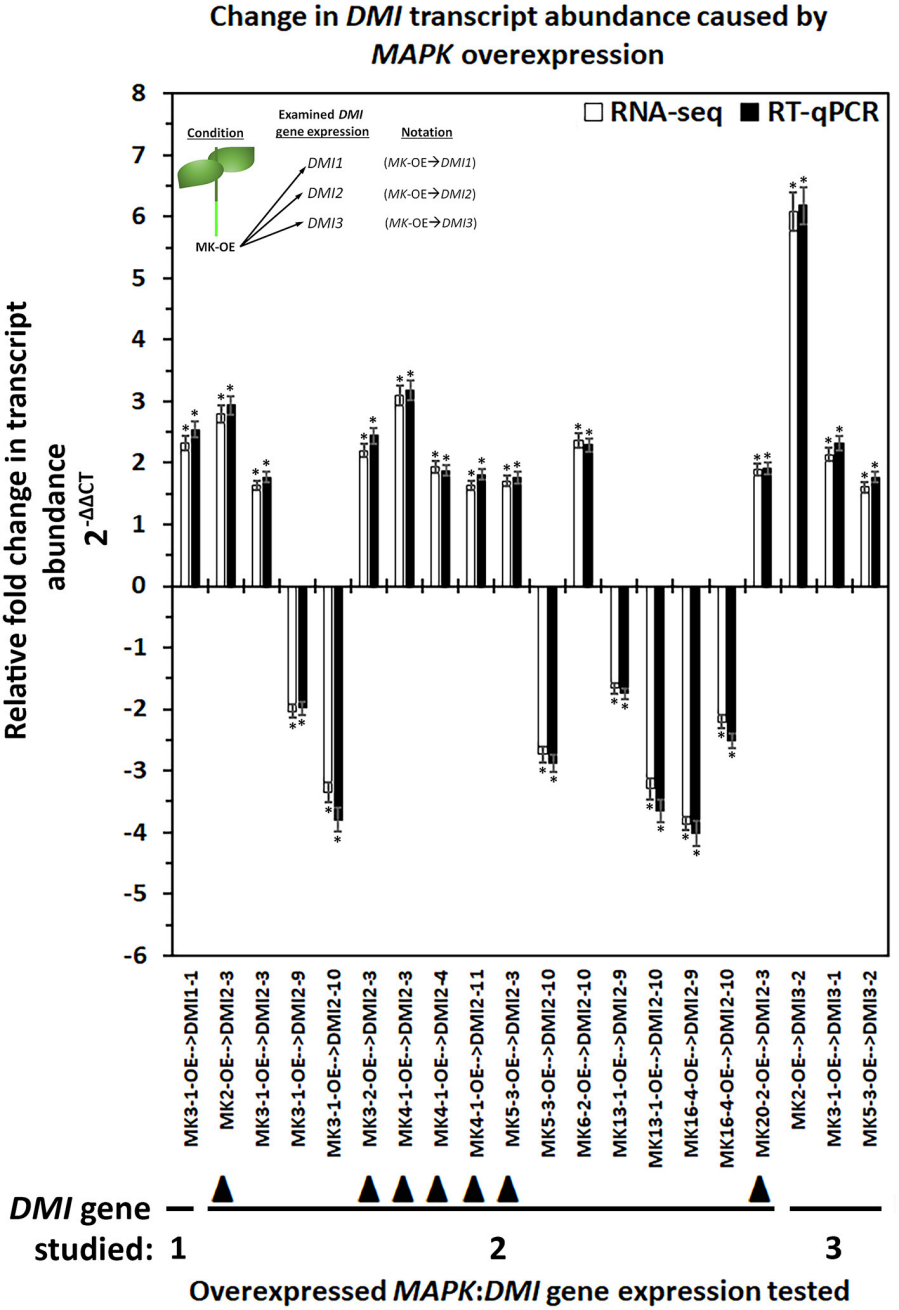
The RTA results, presented as FC, for the *DMI1, DMI2*, and *DMI3* gene family members that had exhibited differential expression in the defense *MAPK*-OE roots measured by RNA-seq and confirmed by RT-qPCR (Livak and Schmittgen, 2001; Wang and Wang, 2021). The data presented in this figure analyzes the *MAPK*-OE root RNA by RNA-seq and RT-qPCR. The sample analysis type presented below the x-axis provides the analysis parameter as *MAPK*-OE with the presented gene expression for the specific *DMI* gene denoted after the arrow ( → ) giving the sample designation as *MAPK*(*specific gene*)-OE → *DMI*(*specific gene*). (*) The RNA-seq result was considered statistically significant at *p* < 0.001, Student's *t*-test; RT-qPCR expression was calculated by 2^−ΔΔCT^ to enumerate the RTA, presented as FC in the y-axis (Livak and Schmittgen, 2001; Klink et al., 2021a). (*) The result is statistically significant at *p* < 0.05, Student's *t*-test (Yuan et al., 2006). The *DMI* genes that had contrasting expression (an FC that was *increased* [I] in the *MAPK*-OE RNA-seq and RT-qPCR analyses (Figure), and an FC that was *decreased* [D] in *MAPK*-RNAi RNA-seq, and RT-qPCR analyses (Figure 4) have been referred to as an *MAPK*-OE:RNAi couplet as designated in the Results subsection: Cross-comparative analyses involving *MAPK*-OE and *MAPK*-RNAi experiments) have been indicated with the designated black triangle (▴). This designation was made for *MAPK2*-OE:RNAi → *DMI2-3* (I:D), *MAPK3-2*-OE:RNAi → *DMI2-3* (I:D), *MAPK4-1*-OE:RNAi → *DMI2-3* (I:D), *MAPK4-1*-OE:RNAi → *DMI2-4* (I:D), *MAPK4-1*-OE:RNAi → *DMI2-11* (I:D), *MAPK5-3*-OE:RNAi → *DMI2-3* (I:D), and *MAPK20-2*-OE:RNAi → *DMI2-3* (I:D). (Figure) presents data for 20 couplets. Details for these experiments can be found in Supplementary Table 6. Please refer to the Materials and Methods subsections: RNA-seq gene expression analyses of transgenic *MAPK* -OE and -RNAi root RNA, and Real-time quantitative PCR (RT-qPCR), for details. Inset, upper left corner of the histogram provides that flow chart that relates to the sample naming scheme found at the x-axis.

**Figure 4:**
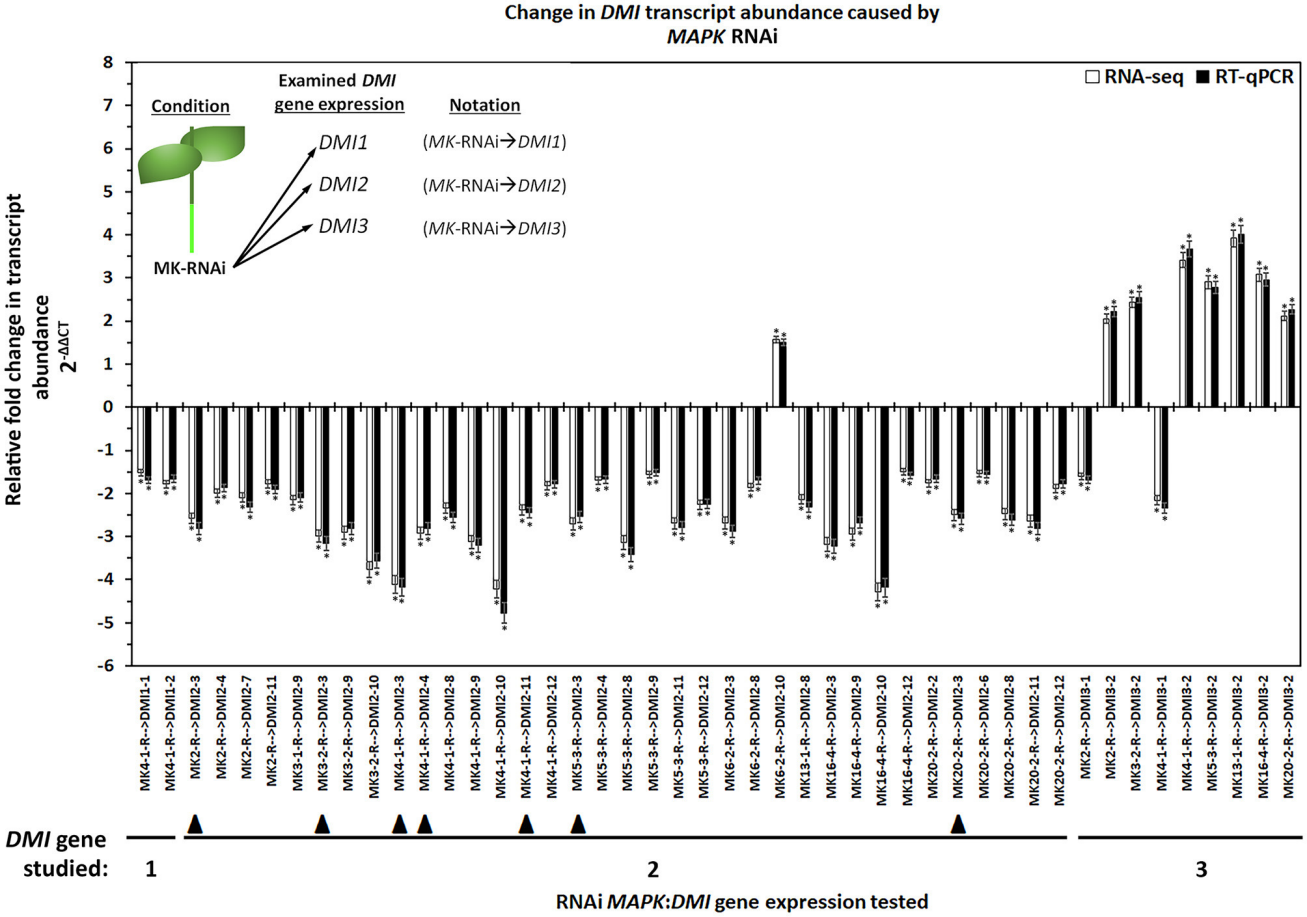
The results for the *DMI1, DMI2*, and *DMI3* gene family members that had exhibited differential expression in the defense *MAPK*-RNAi roots measured by RNA-seq and confirmed by RT-qPCR (Livak and Schmittgen, 2001; Wang and Wang, 2021). The data presented in (Figure) analyzes the *MAPK*-RNAi root RNA by RNA-seq and RT-qPCR. The sample analysis type presented below the x-axis provides the analysis parameter as *MAPK*-RNAi with the presented gene expression for the specific *DMI* gene denoted by an arrow ( → ) giving *MAPK*(*specific gene*)-RNAi → *DMI*(*specific gene*). (*) The RNA-seq result is statistically significant at *p* < 0.001, Student's *t*-test. RT-qPCR expression was calculated by 2^−ΔΔCT^ to enumerate the RTA as presented as the FC (Livak and Schmittgen, 2001; Klink et al., 2021a). (*) RT-qPCR result statistically significant at *p* < 0.05, Student's *t*-test (Yuan et al., 2006). The *DMI* genes that had contrasting expression (an FC that was *increased* [I] in the *MAPK*-OE RNA-seq and RT-qPCR analyses (Figure 3), and an FC that was *decreased* [D] in *MAPK*-RNAi RNA-seq, and RT-qPCR analyses (Figure 4), referred to as an *MAPK*-OE:RNAi couplet as designated in the Results subsection: Cross-comparative analyses involving *MAPK*-OE and *MAPK*-RNAi experiments) were indicated with a black triangle (▴). An arrow ( → ) denotes the *DMI* gene whose expression was examined in relation to the *MAPK*-OE:RNAi RNA-seq and RT-qPCR analyses. This designation was made for *MAPK2*-OE:RNAi → *DMI2-3* (I:D), *MAPK3-2*-OE:RNAi → *DMI2-3* (I:D), *MAPK4-1*-OE:RNAi → *DMI2-3* (I:D), *MAPK4-1*-OE:RNAi → *DMI2-4* (I:D), *MAPK4-1*-OE:RNAi → *DMI2-11* (I:D), *MAPK5-3*-OE:RNAi → *DMI2-3* (I:D) and *MAPK20-2*-OE:RNAi → *DMI2-3* (I:D). This figure presents data for 46 couplets. Details have been provided in Supplementary Table 7. Please refer to Materials and Methods subsections: RNA-seq gene expression analyses of transgenic *MAPK*-OE and RNAi root RNA, and Real-time quantitative PCR (RT-qPCR), for details. Inset, upper left corner of the histogram provides that flow chart that relates to the sample naming scheme found at the x-axis.

In *G. max, MAPK* gene expression had occurred in syncytia undergoing parasitism by *H. glycines* but while undergoing a defense response. These syncytium-expressed defense *MAPK* genes included *MAPK2, MAPK3-1, MAPK4-1, MAPK6-2, MAPK13-1, MAPK16-4*, and *MAPK20-2*. Two other *MAPK*s, *MAPK3-2*, and *MAPK5-3*, whose expression in syncytia that underwent the defense process could not be confirmed due to the original analysis procedures which lacked probe sets on the microarray, had also functioned in defense. RNA-seq data was available for *G. max* defense *MAPK*-OE and *MAPK*-RNAi roots, which included *MAPK2, MAPK3-1, MAPK3-2, MAPK4-1, MAPK5-3, MAPK6-2, MAPK13-1, MAPK16-4*, and *MAPK20-2* as well as their OE (pRAP15-*ccd*B) and RNAi (pRAP17-*ccd*B) controls, respectively. The data was examined here for RTAs that occurred among the parologous members of the *DMI1, DMI2*, and *DMI3* gene families (Supplementary Tables 7, 8). RT-qPCR confirmed the identified *DMI1, DMI2*, and *DMI3* gene family member RTAs that exhibited differential expression in the *MAPK*-OE RNA-seq analyses as compared to the control (Figure 3). There were 153 *MAPK*-OE and *MAPK*-RNAi comparisons made between the 3 different *DMI1* paralogs (*n* = 27), 12 different *DMI2* paralogs (*n* = 108), and 2 different *DMI3* paralogs (*n* = 18) (Supplementary Tables 7, 8). RNA-seq experiments of RNA that was isolated from the *MAPK*-OE roots had identified 20 instances of differentially expressed *DMI* genes that met the set criteria as compared to the control (Figure 3). Please refer to the figure legend and figure inset for the explanation of the examined RNA sample and tested *DMI* gene name designations found at the x-axis. The RTAs of those *DMI* genes as compared to their control were confirmed by RT-qPCR (Figure 3). RNA-seq data was also available for the 9 defense *MAPK*s undergoing RNAi. RNA-seq experiments of RNA isolated from the *MAPK*-RNAi roots identified 46 instances of differentially expressed *DMI* genes that met the set criteria as compared to their control (Figure 4). Please refer to the figure legend and figure inset for the explanation of the name designations found at the *X*-axis. Among the identified *DMI* genes that were found in the *MAPK*-OE analyses was one instance, *DMI1-1*, that had an altered RTA caused by *MAPK3-1* OE that met the set criteria as compared to its control (Figure 3). There were 16 instances of a *DMI2* gene, that involved *DMI2-3, DMI2-4, DMI2-9, DMI2-10*, and *DMI2-11*, that had an altered RTA caused by *MAPK* OE that met the set criteria as compared to its control (Figure 3). There were three instances of a *DMI3* gene, that involved *DMI3-1*, and *DMI3-2*, that had an altered RTA caused by *MAPK* OE that met the set criteria as compared to its control (Figure 3). In contrast, there were 2 instances of a *DMI1* gene, *DMI1-1*, and *DMI1-2*, that had an altered RTA that was caused by *MAPK* RNAi that met the set criteria as compared to its control (Figure 4). There were 35 instances of a *DMI2* gene, that involved *DMI2-3, DMI2-4, DMI2-6, DMI2-7, DMI2-8, DMI2-9, DMI2-10, DMI2-11*, and *DMI2-12*, that had an altered RTA caused by *MAPK* RNAi that met the set criteria as compared to its control (Figure 4). There were 9 instances of a *DMI3* gene, that involved *DMI3-1* and *DMI3-2*, that had an altered RTA caused by *MAPK* RNAi that met the set criteria as compared to its control (Figure 4).

### Cross-Comparative Analyses Made Between the *MAPK*-OE and *MAPK*-RNAi Experiments

**Figure 5:**
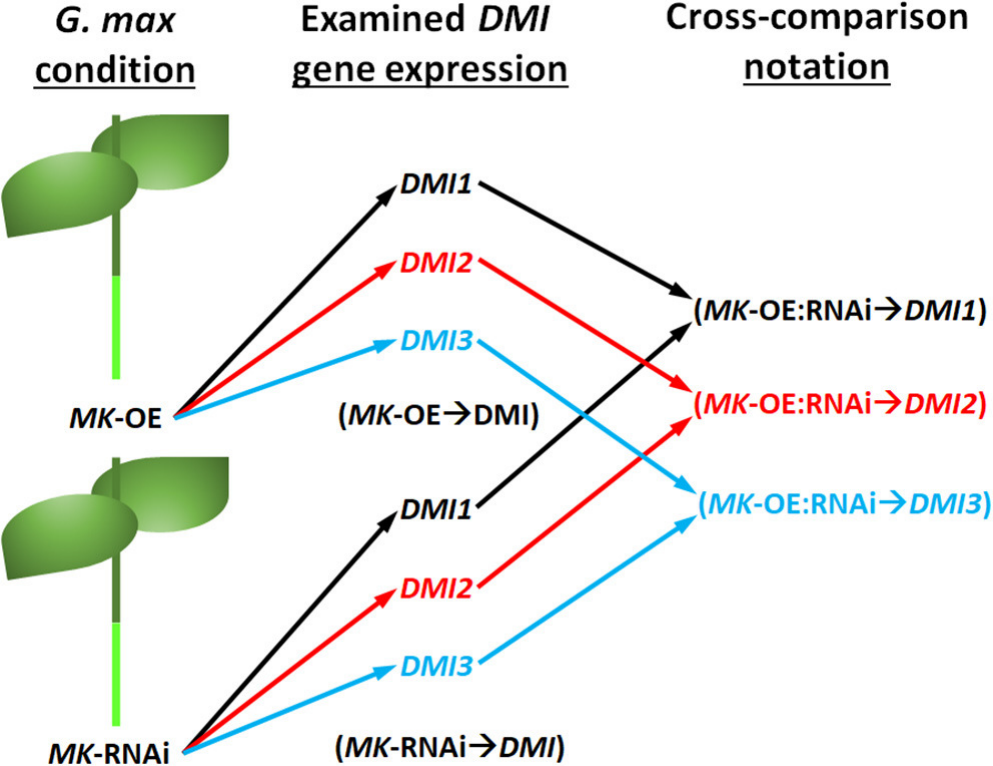
Flow chart of the cross comparison analyses made that used data presented in Figures 3, 4. The condition represents the *MAPK* (*MK*) -OE or -RNAi sample under study. The examined *DMI* gene expression represents the various examined *DMI1, DMI2*, or *DMI3* paralogs. The black, red, and blue arrows and text were used to distinguish between the different *DMI* (*DMI1, DMI2*, and *DMI3*) genes. The notation of the cross comparisons took into consideration that data from both the *MK*-OE and *MK*-RNAi analyses were used in examining the *DMI* gene expression (i.e., *MK*-OE:RNAi → *DMI*).

Cross-comparative analyses were done between the *MAPK*-OE, and *MAPK*-RNAi experiments. The analyses were done to determine whether the experimentally-altered *MAPK* expression (OE or RNAi) exerted contrasting effects on *DMI* RTAs as compared to their controls as might be expected (i.e., increased RTA in the OE sample and decreased RTA in the RNAi sample). Seven instances, referred to as couplets, were identified where the defense *MAPK*-OE increased the *DMI* RTA, while RNAi of that same *MAPK* led to a decreased *DMI* RTA as compared to their controls (Figures 3, 4). All of these instances involved the plasma membrane receptor *DMI2*. To describe these outcomes, the *DMI* genes that had contrasting expression (an FC that was increased [I] in the *MAPK*-OE RNA-seq and RT-qPCR analyses (Figure 3), and an FC that was decreased [D] in *MAPK*-RNAi RNA-seq and RT-qPCR analyses (Figure 4) had been referred to as an *MAPK*-OE:RNAi couplet, indicated with a black triangle [see figure legend]). A flow chart representing the analysis parameters that merge (cross-compare) the OE and RNAi analyses has been provided (Figure 5). An arrow ( → ) was used to denote the *DMI* gene whose expression was examined in relation to the *MAPK*-OE:RNAi RNA-seq and RT-qPCR analyses. This designation was made for *MAPK2*-OE:RNAi → F0E0*DMI2-3* (I:D), *MAPK3-2*-OE:RNAi → F0E0*DMI2-3* (I:D), *MAPK4-1*-OE:RNAi → F0E0*DMI2-3* (I:D), *MAPK4-1*-OE:RNAi → F0E0*DMI2-4* (I:D), *MAPK4-1*-OE:RNAi → F0E0*DMI2-11* (I:D), *MAPK5-3*-OE:RNAi → F0E0*DMI2-3* (I:D) and *MAPK20-2*-OE:RNAi → F0E0*DMI2-3* (I:D) (Figures 3, 4). No instances existed under these conditions where defense *MAPK* OE decreased the *DMI* RTA, and *MAPK* RNAi increased the *DM*I RTA which was shown to happen in Type 1 expression found for some *MAPK*s (McNeece et al., 2019). In McNeece et al. (2019) Type 1 expression spanned the results of both the *MAPK*-OE and -RNAi experiments and was defined that Type 1-OE genes were induced (in their RTA [FC]) in the OE treatment, and were also suppressed in the RNAi treatment or suppressed in the OE treatment, and also induced in the RNAi treatment. Regarding the RNAi experiments, Type 1-RNAi expression was suppressed (in their RTA [FC]) in the RNAi treatment and induced in the OE treatment or induced in the RNAi treatment, and also suppressed in the OE treatment. There were 6 instances identified here whereby the *DMI* gene was differentially expressed in the same manner (increased, decreased) in the *MAPK -*OE, and -RNAi roots as compared to the control under the set criteria. Three of these instances involved increased *DMI* gene RTAs in the *MAPK*-OE, and -RNAi roots. Perhaps importantly, differences in the magnitude of these *DMI* RTAs were observed between the OE, and RNAi experiments and presented subsequently for clarity. The first occurrence was *MAPK2*-OE:RNAi → F0E0*DMI3-2* that exhibited a lower RTA in the *DMI3-2*-RNAi samples that met the set criteria as compared to the controls (Figures 3, 4). The second occurrence was *MAPK5-3*-OE:RNAi → F0E0*DMI3-2* that exhibited higher RTAs in the *MAPK5-3*-RNAi samples that met the set criteria as compared to the controls (Figures 3, 4). The third instance was *MAPK6-2*-OE:RNAi → F0E0*DMI2-10*, that exhibited lower RTAs in the *MAPK6-2*-RNAi samples which met the set criteria as compared to the controls (Figures 3, 4).

Three instances involved decreased *DMI* RTAs as compared to the respective controls. The first instance was *MAPK3-1*-OE:RNAi → F0E0*DMI2-9*, that had about the same RTAs between the *DMI2-9 -*OE, and -RNAi samples and met the set criteria as compared to the controls (Figures 3, 4). The second instance was *MAPK16-4*-OE:RNAi → F0E0*DMI29*, that had lower RTAs in the *MAPK16-4-*RNAi samples, which met the set criteria as compared to the controls (Figures 3, 4). The third instance was *MAPK16-4*-OE:RNAi → F0E0*DMI2-10*, which had higher RTAs in the *MAPK16-4*-RNAi samples that met the set criteria as compared to the controls (Figures 3, 4).

The remaining *DMI* genes satisfied the set differential expression criteria in one of the two conditions (OE or RNAi) but not both, in the manner that Type 2 expression had been described in this pathosystem in McNeece et al. (2019) but for *MAPK*s (Figures 3, 4). In McNeece et al. (2019), Type 2 expression spanned the results of both OE and RNAi experiments. Type 2-OE was expression that was induced (in their RTA [FC]) in the OE treatment, and not differentially expressed in the RNAi treatment or was suppressed in the OE treatment, and not differentially expressed in RNAi treatment (McNeece et al., 2019). Within Type 2 expression, Type 2-RNAi had suppressed expression in the RNAi treatment while it was not differentially expressed in the OE treatment or was induced in the RNAi treatment and was not differentially expressed in the OE treatment (McNeece et al., 2019). An examination of *MAPK*-OE data was made that resulted in the identification that *DMI1* had one case (*DMI1-1*) of an altered RTA but in a *MAPK*-OE (*MAPK3-1*-OE) sample, and which met the set criteria as compared to the controls (Figure 3). *DMI2* had 9 cases involving *DMI2-3, DMI2-4, DMI2-9, DMI2-10*, and *DMI2-11* of an altered RTA but in a *MAPK*-OE, and which met the set criteria as compared to the controls (Figure 3). *DMI3* had 3 cases that involved *DMI3-1*, and *DMI3-2* of an altered RTA but in a *MAPK*-OE, and which met the set criteria as compared to the controls (Figure 3). In contrast, an examination of *MAPK*-RNAi RNA-seq data was made. *DMI1* had 2 cases (*DMI1-1*, and *DMI1-2*) of altered RTAs, that occurred in the *MAPK4-1*-RNAi sample, and which met the set criteria as compared to the controls (Figure 4). *DMI2* had 28 cases that involved *DMI2-*2, *DMI2-3, DMI2-4, DMI2-7, DMI2-8, DMI2-9, DMI2-10, DMI2-11*, and *DMI2-12* that had an altered RTA but occurred in an examined *MAPK*-RNAi sample, and which met the set criteria as compared to the controls (Figure 4). *DMI3* had 9 cases that involved *DMI3-1*, and *DMI3-2* that had an altered RTA but occurred in an examined *MAPK*-RNAi sample and met the set criteria as compared to the controls (Figure 4). These cases included the *MAPK2*-RNAi, *MAPK3-2*-RNAi, *MAPK4-1*-RNAi, *MAPK5-3*-RNAi, *MAPK13-1*-RNAi, *MAPK16-4*-RNAi, and *MAPK20-2*-RNAi samples (Figure 4). Other examples of differentially expressed *DMI* genes that were identified in the defense *MAPK* RNA-seq analyses existed, but they had not met the set FC criteria, and were not examined further (Supplementary Tables 7, 8). However, their expression could be important and potential targets of future analyses.

### The Identification of *MAPK*-Altered *DMI* Expression to Below Experimental-Threshold Levels

The RTAs of *DMI1-3, DMI2-7*, and *DMI3-2* used in the functional transgenic studies that examined *H. glycines* infection were identified in the *MAPK*-OE, and *MAPK*-RNAi RNA-seq experiments as compared to their respective transgenic pRAP15-*ccd*B and pRAP17-*ccd*B controls (Table 3 and Supplementary Tables 7, 8). A number of instances of statistically significant changes in *DMI* RTAs were noted but occurred below the set FC criteria. Therefore, while noteworthy were beyond the scope of further analysis here. Generally, the *DMI* RTAs that occurred in the RNA-seq analyses were lower than those found for the *DMI1-3, DMI2-7*, or *DMI3-2* -OE or -RNAi roots in the RT-qPCR quality control analyses of the root RNA under comparison in the functional transgenic infection studies.

**Table 3:** RNA-seq differential expression measurements of *G. max DMI-3, DMI2-7*, and *DMI3-2* gene family members in the defense *MAPK*-OE and *MAPK*-RNAi root RNA samples as compared to the pRAP15*-ccdB* (OE control) and pRAP17*-ccdB* (RNAi control) samples, *p* < 0.001 (Wang and Wang, 2021).

**Condition**	**Gene expression**	***MK*-OE → *DMI* FC**	***MK*-RNAi → *DMI* FC**
	**Examined** ***DMI***		
*MK2*-OE:RNAi	*DMI1-3*	−0.509168236	−0.606009451
*MK3-1*-OE:RNAi	*DMI1-3*	nde	−0.258663037
*MK3-2*-OE:RNAi	*DMI1-3*	nde	0.33078036
*MK4-1*-OE:RNAi	*DMI1-3*	nde	−0.64650619
*MK5-3*-OE:RNAi	*DMI1-3*	nde	0.45370847
*MK6-2*-OE:RNAi	*DMI1-3*	nde	nde
*MK13-1*-OE:RNAi	*DMI1-3*	nde	0.987644973
*MK16-4*-OE:RNAi	*DMI1-3*	nde	nde
*MK20-2*-OE:RNAi	*DMI1-3*	nde	0.548846371
*MK2-OE*:RNAi	*DMI2-7*	0.751995656	−2.094693822
*MK3-1*-OE:RNAi	*DMI2-7*	nde	nde
*MK3-2*-OE:RNAi	*DMI2-7*	nde	−0.991278883
*MK4-1*-OE:RNAi	*DMI2-7*	nde	−1.481257961
*MK5-3*-OE:RNAi	*DMI2-7*	nde	−1.320032574
*MK6-2*-OE:RNAi	*DMI2-7*	nde	−0.578103045
*MK13-1*-OE:RNAi	*DMI2-7*	nde	nde
*MK16-4*-OE:RNAi	*DMI2-7*	1.158304234	−1.189694562
*MK20-2*-OE:RNAi	*DMI2-7*	1.28018865	−0.863199158
*MK2*-OE:RNAi	*DMI3-2*	6.082764374	2.052151835
*MK3-1*-OE:RNAi	*DMI3-2*	2.131484967	1.405468239
*MK3-2*-OE:RNAi	*DMI3-2*	nde	2.434281191
*MK4-1*-OE:RNAi	*DMI3-2*	nde	3.417301738
*MK5-3*-OE:RNAi	*DMI3-2*	1.601640755	2.903015546
*MK6-2*-OE:RNAi	*DMI3-2*	nde	1.004285599
*MK13-1*-OE:RNAi	*DMI3-2*	nde	3.916486594
*MK16-4*-OE:RNAi	*DMI3-2*	nde	3.074298912
*MK20-2*-OE:RNAi	*DMI3-2*	1.252369636	2.119541855

Terminology: relative transcript abundance (RTA); fold change (FC); not differentially expressed (nde); overexpression (OE); RNA interference (RNAi). The defense MAPK-OE RNA-seq data were compared to the pRAP15-ccdB OE control RNA-seq data that were generated and analyzed as stated in the Materials and Methods subsections: G. max genetic transformations, and RNA-seq gene expression analyses of transgenic MAPK-OE and RNAi root RNA, respectively. The defense MAPK-RNAi RNA-seq data were compared to the pRAP17-ccdB RNAi control RNA-seq data generated and analyzed as stated in the Materials and Methods subsection: G. max genetic transformations, and RNA-seq gene expression analyses of transgenic MAPK-OE and RNAi root RNA, respectively. ([^*^] statistically significant with a FC > ± 1.5; p < 0.001) (Wang and Wang, 2021). The change in MAPK-OE RTA was presented as a FC measured between the MAPK-OE RNA-seq data as compared to the pRAP15-ccdB control RNA-seq data (Wang and Wang, 2021). The change in MAPK-RNAi RTA was presented as a change in the FC that occurred between the analysis of the MAPK-RNAi RNA-seq data as compared to the pRAP17-ccdB control RNA-seq data (Wang and Wang, 2021). Note, these MAPK-OE, MAPK-RNAi, pRAP15-ccdB and pRAP17-ccdB roots were used in functional transgenic H. glycines infection analyses that determined their respective female index (FI) with RNA-seq analyses performed on isolated RNA (Alshehri et al., 2019; McNeece et al., 2019). Note, the description of the condition is the same as described in Figures 3, 4. The description was provided here for added clarity. The data presented in table analyzed the DMI gene (DMI1-3, DMI2-7, or DMI3-2) whose OE was examined in the RNA-seq samples for the MAPK -OE or MAPK -RNAi RNA-seq data. The condition provided the analysis parameter as MAPK (MK) -OE with the presented gene expression for the specific DMI gene denoted after the arrow ( → ) giving the sample designation as MK(specific gene)-OE → DMI(specific gene). (^*^) The RNA-seq result was considered statistically significant at p < 0.001, Student's t-test. The same analysis was done for the MK -RNAi. The RNAi condition provided the analysis parameter as MK-RNAi with the presented gene expression for the specific DMI gene denoted after the arrow ( → ) giving the sample designation as MK(specific gene)-RNAi → DMI(specific gene). (^*^) The RNA-seq result was considered statistically significant at p < 0.001, Student's t-test.

### Altered *MAPK3* RTAs Occur in the *DMI1, DMI2*, and *DMI3* Transgenic Roots

**Figure 6:**
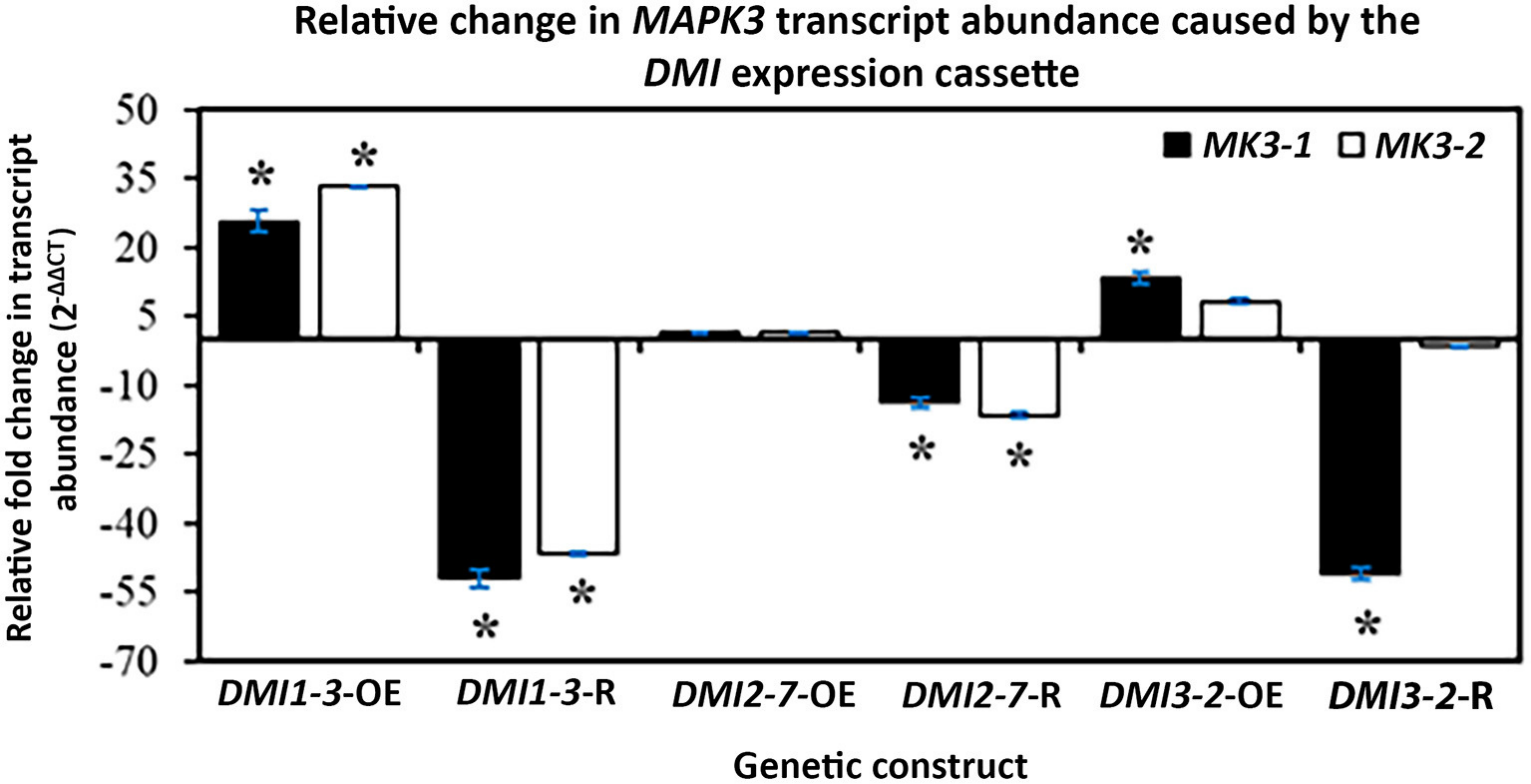
The effect that *DMI1, DMI2*, and *DMI3* -OE or -RNAi had on the RTA, presented as FC, of *MAPK3-1*, and *MAPK3-2*, confirmed by RT-qPCR as calculated by 2^−ΔΔCT^ (Livak and Schmittgen, 2001). (*) *p* < 0.05, Student's *t*-test (Yuan et al., 2006). Please refer to Materials and Methods subsection: Real-time quantitative PCR (RT-qPCR), for details.

The MAPK3 protein transduces signals from both PTI and ETI branches of defense signaling that leads to an output response that combats pathogen infection including parasitism by pathogenic nematodes. RT-qPCR experiments that targeted *MAPK3-1* and *MAPK3-2* were done that determined if genes functioning to transduce defense signaling through PTI and ETI were affected by OE, and/or RNAi of the syncytium-expressed *DMI1-3, DMI2-7*, and *DMI3-2*. Those results that employed RT-qPCR have been presented (Figure 6). The gene expression experiments showed *DMI1-3*-OE increased *MAPK3-1* and *MAPK3-2* RTA while, in contrast, *DMI1-3*-RNAi decreased *MAPK3-1*, and *MAPK3-2* RTA as compared to the controls (Figure 6). *DMI2-7*-OE did not significantly affect *MAPK3-1* or *MAPK3-2* RTA, while *DMI2-7*-RNAi significantly decreased both *MAPK3-1* and *MAPK3-2* RTA as compared to the controls (Figure 6). *DMI3-2* -OE increased *MAPK3-1* RTA but had no effect on *MAPK3-2* RTA as compared to the controls (Figure 6). *DMI3-2*-RNAi decreased *MAPK3-1* RTA but had no effect on *MAPK3-2* RTA as compared to the controls (Figure 6). The results indicate *DMI1-3, DMI2-7*, and *DMI3-2* had functions in common with 2 major *G. max* defense genes (*MAPK3-1*, and *MAPK3-2*) that act in PTI and ETI, but that there are important differences possibly relating to the unique functions that the *DMI* genes have in relation to the respective *MAPK3-1*, and *MAPK3-2* paralogs. The results provide clear evidence that *DMI1-3, DMI2-7*, and *DMI3-2* influence the RTAs of *MAPK* genes that function downstream during PTI and ETI. The unique functions of the *DMI* paralogs as it relates to *MAPK* gene expression and gene expression in general are discussed subsequently.

## Discussion

The capability of EPNs to impair symbioses indicates the molecular signaling machinery responses associated with symbiosis and pathogenicity are intertwined at some level. The knowledge of different *DMI* genes functioning at different points in the CSP and the expression of *G. max* homologs occurring within syncytia undergoing a defense response were a reasonable place to start an analysis. General aspects of plant signaling are accomplished through a number of basic pathways. Among these pathways is MAPK signaling, and several lines of evidence link symbiosis to MAPK signaling. Analyses in the *G. max*-*H. glycines* pathosystem demonstrate the importance of MAPK signaling to its defense response so it is examined further here in relation to the *DMI* genes.

### Identification of *G. max DMI* Genes

The experimental analysis presented here explores a prior observation of *DMI3* expression occurring in syncytia undergoing the defense process to *H. glycines* parasitism (Klink et al., 2010b). Analyses presented here reveal that the *DMI3* identified in Klink et al. (2010b) is *DMI3-2*. The result indicates that genes acting in the CSP may also function in defense.. The experiments presented here focus in on a specific subset of CSP genes, *DMI1, DMI2*, and *DMI3*. *G. max* homologs of *DMI1* (3 paralogs), *DMI2* (12 paralogs), and *DMI3* (2 paralogs), while splice variants are also identified. Analyses of other genomes of select agriculturally important crops identify their *DMI1, DMI2*, and *DMI3* gene families, and splice variants. The examined genomes have between 1 and 7 (or more depending on stringency) *DMI1* paralogs with the hexaploid *T. aestivum* having by far the most (7). The examined genomes have between 0 and 3 *DMI2* and *DMI3* paralogs with *B. rapa* (rape seed) and *B. vulgaris* (sugarbeet) having none. *A. thaliana* lacks *DMI3*, thought to explain one of the reasons why it cannot establish symbiosis with AM fungi (Zhu et al., 2006). This observation may also partially explain their absence in *B. rapa* and *B. vulgaris*. Splice variants of conserved genes are important in the plant defense process (Bazin et al., 2020). Alternative spliced transcripts are present within the syncytium and are an important feature that *G. max* employs in its defense response toward *H. glycines* infection (Lawaju et al., 2020; Klink et al., 2021b). The bioinformatics analyses presented here also identify numerous splice variants of the *DMI* genes. Some of the genomes, in particular *T. aestivum*, exhibit large numbers of predicted splice variants, their nature requiring functional experimentation to determine a biological role.

### *DMI* Genes Are Expressed in Root Cells Undergoing a Defense Function

Since a goal here is to examine the function of the *G. max DMI* genes, comparisons to syncytium gene expression data leads to the confirmation of one paralog of each gene family being expressed within the syncytium undergoing the defense response. This observation allows for narrowing down the number of studied paralogs to one per gene family. However, the expression of a number of *DMI* genes are not determined because probe sets for those genes do not exist on those *G. max* microarrays and, thus, require further analysis beyond this works' scope. The functional examination of genes expressed in root cells undergoing defense to *H. glycines* is a sound approach to identify genes that function in impairing its parasitism (Matsye et al., 2011, 2012; McNeece et al., 2019; Klink et al., 2021a). Consequently, the *DMI1-3, DMI2-7*, and *DMI3-2* genes have been cloned and engineered here for OE and RNAi into *G. max* in the targeted genotypes for functional transgenic studies as compared to their respective controls. Transgenic OE and RNAi of *DMI1-3, DMI2-7*, and *DMI3-2* lead to the expected increase in their RTAs while their RNAi leads to their expected decreased RTAs. The effect that the *DMI1-3, DMI2-7*, and *DMI3-2* OE and RNAi cassettes have on expression is further shown here to continue to occur at 6 dpi at a time point when the natural defense response has concluded. The effect that the *DMI1-3, DMI2-7*, and *DMI3-2* OE and RNAi cassettes have on *H. glycines* parasitism as compared to their respective controls (discussed in the next section) are reflective of their expression within syncytia undergoing a defense response. Among the transgenic experiments, *DMI1-3* OE leads to a 2.1-fold increase in root mass. This observation is not surprising since the stimulation of DMI1 and DMI2 signaling in *M. truncatula* results in an increase in lateral root formation (Oláh et al., 2005).

### Functional Transgenic Analyses Reveal a Defense Role for *G. max DMI* Genes

The functional transgenic studies are the OE and RNAi of the *DMI1-3, DMI2-7*, and *DMI3-2* genes as compared to their relevant transgenic controls. The analyses then follow infection by *H. glycines* during a 30 day time course, leading to the quantification of their effect on parasitism as measured through the FI. The expected experimentally-affected increase in *DMI* RTA in the OE roots is observed as compared to the controls, as demonstrated by RT-qPCR of RNA sample template isolated from in the *H. glycines*-susceptible *G*.*max*_[Williams82/PI518671]_. In contrast, a decrease in *DMI* RTA is observed as compared to the controls, as demonstrated by RT-qPCR using RNA template isolated from the roots of the *DMI*-RNAi root RNA of the *H. glycines*-resistant *G*.*max*_[Peking/PI548402]_ (Livak and Schmittgen, 2001). These outcomes provide the set criteria required for the infection of transgenic *DMI1-3, DMI2-7*, and *DMI3-2* -OE and -RNAi roots and respective controls by *H. glycines*. Hundreds of gene constructs, experimented on in this pathosystem once these criteria have been met, include genes functioning in the PTI and ETI defense branches which converge on the MAPK signaling platform (McNeece et al., 2019; Klink et al., 2021a). The experimentally-induced expression of *G. max* PTI components including *BRI1-ASSOCIATED RECEPTOR KINASE 1* (*BAK1*) *BAK1-1*, and *BOTRYTIS INDUCED KINASE1* (*BIK1*), *BIK1-6*, and ETI components including the bacterial effector harpin, *NON-RACE SPECIFIC DISEASE RESISTANCE 1* (*NDR1*) *NDR1-1*, and the nitrate-induced (NOI) domain-containing, intrinsically disordered, molecular recognition feature (MoRF)-containing *RPM1-INTERACTING PROTEIN 4* (*RIN4*) *RIN4-4* all increase *MAPK3-1* and *MAPK3-2* RTA and function in the defense response that *G. max* has to *H. glycines* parasitism (Wei et al., 1992; Century et al., 1995, 1997; Gopalan et al., 1996; Li and Chory, 1997; Desikan et al., 2001; Li et al., 2002; Mackey et al., 2002, 2003; Day et al., 2006; Veronese et al., 2006; Pant et al., 2014; Sun et al., 2014; Aljaafri et al., 2017; McNeece et al., 2017, 2019; Klink et al., 2021a). The results of enumerating *H. glycines* from the transgenic roots demonstrates that the OE of *DMI1-3, DMI2-7*, and *DMI3-2* genes leads to a significant decrease in *H. glycines* parasitism as compared to the pRAP15-*ccd*B OE control in the otherwise *H. glycines*-susceptible *G*.*max*_[Williams82/PI518671]_ genetic background. In contrast, RNAi of *DMI1-3, DMI2-7*, and *DMI3-2* genes, leads to an increase in *H. glycines* parasitism as compared to the transgenic pRAP17-*ccd*B RNAi control in the otherwise normally *H. glycines*-resistant *G*.*max*_[Peking/PI548402]_ genetic background. The combination of decreased parasitism in the otherwise normally *H. glycines*-susceptible genetic background *G*.*max*_[Williams82/PI518671]_ and increased parasitism in the otherwise normally *H. glycines*-resistant *G*.*max*_[Peking/PI548402]_ genetic background are taken as evidence that the gene functions in the defense response because of the ability to obtain these opposite outcomes that are caused by target gene OE and RNAi (Pant et al., 2014; Sharma et al., 2016; McNeece et al., 2019; Klink et al., 2021a). *DMI1, DMI2*, and *DMI3* function at different stages of the CSP (Catoira et al., 2000; Ané et al., 2002, 2004). This observation is similar to those made by McNeece et al. (2019) for the MAPK defense signaling pathway. Therefore, the CSP, containing the *DMI* genes, appears to function in complex ways during the defense process and whose understanding may benefit by examining their gene regulation in relation to MAPK signaling (Francia et al., 2011).

The determination that the *G. max DMI* genes function in complex ways began with the observation that a large difference exists in the *DMI1-3*-OE pg FI analysis as compared to the wr analysis. As stated, these observations are not surprising since stimulation of DMI1 and DMI2 signaling in *M. truncatula* results in an increase in lateral root formation which would be expected to lead to an increase in root mass (Oláh et al., 2005). The observation that the CCaMK DMI3 has a defense role is consistent with observations that a *G. max* calmodulin (Glyma.19G068300) also functions in the defense process (Matthews et al., 2013). With the demonstration that *DMI1-3, DMI2-7*, and *DMI3-2* genes function in defense, further characterization of the genes began in order to understand the possible function(s) that they may have in signaling.

### MAPKs and Symbiosis

*Lupinus albus* (white lupine) is capable of undergoing a salt-induced stress response that leads to the expression of *MAPK*s (Fernandez-Pascual et al., 2006). Some of these MAPKs are activated (phosphorylated) after host infection with the nodule-inducing *Bradyrhizobium* sp. (Fernandez-Pascual et al., 2006). The MAPK activation process happens in the root's infection zone and is impaired by MAPK inhibitors, indicating a positive role that MAPKs have in this nodulation process (Fernandez-Pascual et al., 2006). MAPKK inhibitors also alter the nodulation pattern (Fernandez-Pascual et al., 2006). The altered nodulation includes decreasing the number and mass of nodules in the upper root while increasing them in the lower register of the root zone, with MAPK inhibition blocking early infection events and delaying the nodule developmental process (Fernandez-Pascual et al., 2006).

In contrast, results demonstrate that MAPK signaling can also interfere with nodulation. The *M. truncatula* MtMKK5-MtMPK3/6 signaling module impairs the early symbiotic nodule formation process, and it is believed the MAPK system's role occurs upstream of ERF Required for Nodulation 1 (ERN1) and Nod factor Signaling Pathway 1 (NSP1) (Ryu et al., 2017). MtMKK5 OE in *M. truncatula* stimulates stress and defense signaling pathways, reducing nodule formation (Ryu et al., 2017). Furthermore, treatment with the MAPK U0126 inhibitor enhances nodule formation while increasing the RTA of the early *M. truncatula Nodule Inception nodulation* (*MtNIN*) (Ryu et al., 2017). MtMKK5 directly activates MtMPK3/6, promoting their physical interaction with the early nodulation ERN1 and NSP1 transcription factors (Ryu et al., 2017). The *Sinorhizobium* sp. strain NGR234 Nodulation outer protein L (NopL) is a *Rhizobium*-specific type 3 effector that acts in nodulation (Zhang et al., 2011). Treatment of *Phaseolus vulgaris* (common bean) with strain NGRΩnopL having a mutated nopL leads to the development of ineffective, senescing nodules, indicating that NopL antagonizes nodule senescence (Zhang et al., 2011). NopL interferes with MAPK signaling in *Nicotiana tabacum* (tobacco), suppressing apoptosis induced by the OE of the MAPK gene *Salicylic acid-induced protein kinase* (*SIPK*) OE or constitutive activity caused by a *SIPK*(DD) (mutation in the TXY motif) (Zhang et al., 2011). NopL acts by mimicking a MAPK substrate and by doing so, impairs MAPK signaling which results in suppressing premature nodule senescence (Zhang et al., 2011). A second type 3 secreted effector, the E3 ubiquitin ligase NopM, is shown through *In planta* NopM expression in *N. tabacum* to induce cell death, stimulating effector-triggered immunity responses and NopM expression in *L. japonicus* reduces nodule formation (Xu et al., 2018). Phosphorylation of NopM by the salicylic acid-induced protein kinase (NtSIPK) MAPK occurs *in vitro* (Xu et al., 2018). In *L. japonicus*, Symbiosis receptor-like kinase (SymRK) mediates the legume-*Rhizobium* symbiosis (Yin et al., 2019). SymRK is the ortholog of DMI2, interacting with NFR1 and NFR5 in promoting NF signaling. The MAPKK SIP2 is a SymRK-interacting protein that positively regulates nodule organogenesis (Yin et al., 2019). This result directly links the DMI2 membrane receptor to MAPK signaling. Furthermore, LjMPK6 is a phosphorylation target of SIP2 (Yin et al., 2019). LjMPK6 RNAi decreases nodule primordia (NP) and nodule number, while LjMPK6-OE increases nodule, infection threads (ITs), and NP numbers demonstrating a positive role for LjMPK6 in nodulation (Yin et al., 2019). Further exploration of the LjMPK6 phosphorylation in relation to nodulation identify the LjMPK6-interacting type 2C protein phosphatase, LjPP2C, which dephosphorylates LjMPK6 *in vitro* and *in vivo* in, functioning to fine-tune nodule development after rhizobia inoculation (Yan et al., 2020). Analysis of hairy root transformed plants demonstrate a non-phosphorylatable mutant LjMPK6 (T224A Y226F) mimics LjPP2C, illustrating LjPP2C phosphatase dephosphorylates LjMPK6 to fine tune nodule development in *L. japonicus* (Yan et al., 2020). The upstream SIMK activator SIMK kinase (SIMKK) infected with *Sinorhizobium meliloti* show in *SIMKK* RNAi lines exhibiting downregulation of both SIMKK and SIMK, having reduced root hair growth while also having a decrease in ability to form infection threads and nodules (Hrbáčková et al., 2021). Constitutive SIMK OE promotes root hair growth, infection thread, and nodule clustering (Hrbáčková et al., 2021). The results are consistent with the involvement of *DMI* genes in *G. max* in AM symbioses (Navazio et al., 2007). Furthermore, the results identify a link between *DMI* genes and MAPKs.

Phosphoproteomic analyses made in conjunction with gene expression studies of NF-induced control and *dmi3* mutants identify gene expression changes and phosphorylation events correlating with nodulation (Rose et al., 2012). Consequently, not only do the *DMI* genes function at specific stages of the CSP, but also govern gene expression. A *dmi3*-mediated feedback loop also exists and is present for interactions involving the response to symbiotic AM fungi (Rose et al., 2012). DMI3 plays a role in the epidermis and cortex during symbiosis (Rival et al., 2012). DMI functions in the activation of downstream genes that act in symbiosis (Czaja et al., 2012). Rhizobia and purified NFs induce the expression of *M. truncatula DMI3* leading to the expression of AM symbiosis and nodulation ATP-binding cassette (ABC) B-type transporters including *ABCB for mycorrhization and nodulation1-3* genes (*AMN1, AMN2*, and *AMN3*) (Roy et al., 2021). DMI is also regulated by plant hormones, including abscisic acid (ABA) (Ni et al., 2019). In *O. sativa*, ABA inhibits rice protein phosphatase PP45 which occurs through H_2_O_2_ leading to relieving DMI3 repression (Ni et al., 2019). The inactivation of DMI3 by PP45 occurs by dephosphorylating Thr-263 in DMI3. ABA-induced H_2_O_2_ production occurring through the NADPH oxidases inhibits the PP45 activity of PP45 by inhibiting *PP45* expression and by oxidizing Cys-350 and Cys-428 residues, forming PP45 intermolecular dimers, blocking the interaction between PP45 and DMI3, preventing PP45-mediated DMI3 activity inhibition. DMI3 also plays roles in the regulation of genes whose expression is important to nodulation, including CCAAT-box-binding transcription factors (Laloum et al., 2014). Other downstream genes include *M. truncatula auxin resistant 1/like aux1* (*AUX1*/*LAX*) influx carriers, *LAX* (*MtLAX*), *M. truncatula pin-formed* (*PIN*) efflux carriers (*MtPIN*), and *M. truncatula MtABCB* gene families (Shen et al., 2015). These genes, characterized in roots and shoots, indicate that three representative auxin transporters (MtLAX3, MtPIN7, and MtABCB1) have their plasma membrane localizations up-regulated in the roots by *Sinorhizobium meliloti* infection in the wild type (WT) but down-regulated in both the roots and shoots of *dmi3* (Shen et al., 2015). The impairment of symbiosis by EPNs indicates the processes are antagonistic. Gene expression studies would provide some evidence supporting the hypothesis. The expression of the *G. max DMI3-2* in syncytia experiencing defense is consistent with the observation that symbiosis genes can also have a dual function in defense (Mitra et al., 2004; Klink et al., 2010b). The experiments presented here place those observations into the context of gene defense function, gene expression, and gene co-regulation.

### *MAPK*s Regulate *DMI* Gene Expression to Levels Below the Cutoff Threshold

An examination of the RNA-seq data identifies a lower threshold of *MAPK*-regulated *DMI* expression occurring at a statistically significant level for each *DMI* gene. This description focuses in on the syncytium-expressed *DMI1-3, DMI2-7*, and *DMI3-2* genes. RNA-seq analyses show *DMI1-3* RTAs are affected to statistically-significant levels by RNAi of *MAPK3-1* (FC,−0.6060), *MAPK3-2* (FC,−0.2587), *MAPK4-1* (FC, 0.3308), *MAPK5-3* (FC,−0.6466), *MAPK13-1* (FC, 0.9876), and *MAPK20-2* (FC, 0.5488) while none of the *MAPK* overexpressing roots affect *DMI1-3* RTAs. Low levels of defense gene expression, including syncytium-expressed conserved oligomeric Golgi (COG) complex members, components of the exocyst, and various other genes are reported (Niraula et al., 2020; Sharma et al., 2020; Klink et al., 2021b).

For *DMI2-7, MAPK2*-RNAi leads to a decrease in RTA, confirmed by RT-qPCR and already discussed. However, RNA-seq analyses show that *MAPK2*-OE leads to a statistically significant increase in *DMI2-7* RTA (FC, 0.7520). Therefore, *MAPK2* OE leads to an increase in *DMI2-7* RTA while its RNAi decreases its RTA. *DMI2-7* is also affected by *MAPK16-4*-OE (FC, 1.1583) and *MAPK16-4*-RNAi (FC,−1.1897) *MAPK20-2*-OE (FC, 1.2802) and *MAPK20-2*-RNAi (FC,−0.8632). However, while *MAPK3-1, MAPK3-2, MAPK4-1*, and *MAPK6-2* do not appear to affect *DMI2-7* RTAs, expression of their RNAi cassettes appear to have a negative impact. For example, RNAi of *MAPK3-2* (FC,−0.9913), *MAPK4-1* (FC,−1.4813), *MAPK5-3* (FC,−1.3200), and *MAPK6-2* (FC,−0.5781) appear to decrease *DMI2-7* RTAs.

For *DMI3-2*, the OE of *MAPK2*, and *MAPK5-3* and -RNAi genetic cassettes have opposite effects on their RTA as described. Other effects on *DMI3-2* RTA are caused by *MAPK20-2*-OE (FC, 1.2524), meaning its OE and RNAi have opposite effects as well. Furthermore, *MAPK3-1* RNAi increases *DMI3-2* RTA (FC, 1.4055), but not as much as *MAPK3-1* OE. While *MAPK6-2*-OE has no effect on *DMI3-2* RTA, its RNAi increases its RTA (FC, 1.0043).

### *MAPK3* As It Relates to *DMI* Expression

MAPK3 is one of the most studied MAPKs as it relates to plant defense (Desikan et al., 1998; Asai et al., 2002; McNeece et al., 2019). The expression of each of the *G. max MAPK3* paralogs (*MAPK3-1*, and *MAPK3-2*) in each of the *DMI1-3, DMI2-7*, and *DMI3-2* overexpressing and RNAi lines are examined here to determine whether a relationship exists between them. The results of those analyses show that *DMI1-3* overexpressing roots have an increase in transcript abundance for *MAPK3-1*, and *MAPK3-2*. The results of those analyses also show that *DMI1-3* RNAi roots have a decrease in RTA for *MAPK3-1*, and *MAPK3-2*. Consequently, it is evident that altered *DMI1-3* expression leads to modulated *MAPK3-1*, and *MAPK3-2* expression, linking the two pathways from a transcriptomic level. As stated earlier, DMI1 is a nuclear membrane protein that is necessary for the initiation of NF-induced Ca^2+^ spiking response in root hairs and functions upstream of DMI3, NSP1, and NSP2 (Wais et al., 2000).

An examination of the *DMI2-7*-OE RNA shows no effect on the RTA for *MAPK3-1*, and *MAPK3-2*. In contrast, *DMI2-7* RNAi roots have a decrease in RTA for *MAPK3-1*, and *MAPK3-2*. Therefore, it is clear that decreased *DMI2-7* RTAs leads to suppressed *MAPK3-1*, and *MAPK3-2* expression in the RNAi roots, while no effect is observed in the *DMI2-7*-OE roots. These results also link the DMI and MAPK3 pathways from a transcriptomic standpoint. The observation that *DMI2-7* OE does not affect *MAPK3-1* or *MAPK3-2* RTAs is noteworthy. It is possible that the maximum number of DMI2-7 plasma membrane receptors are already present, achieving maximum sensitivity. Consequently, a further increase through OE does not affect downstream *MAPK3-1* or *MAPK3-2* levels, indicating a possible negative feedback regulation in this case.

An examination of the *DMI3-2* -OE RNA shows an increase of the RTA, but only for *MAPK3-1* while *MAPK3-2* is not affected. In contrast, an examination of the *DMI3-2*-RNAi RNA shows a decrease of the RTA, but only for *MAPK3-1* while *MAPK3-2* is not affected. This result demonstrates that there is a level of specificity in relation to the direction of influence that the DMI pathway has on defense. McNeece et al. (2019) demonstrates that the plasma membrane protein *NDR1*, also known as *HARPIN INDUCED1* (*HIN1*), which can become increased in its RTA by the bacterial effector harpin, leads to co-regulated expression occurring between *MAPK3-1*, and *NDR1*. Furthermore, *MAPK3-1* OE leads to increased RTA of the *Rhg4* gene *serine hydroxymethyltransferase, galactinol synthase* (*GS-3*), *reticuline oxidase* (*RO-40*), and *xyloglucan endotransglycosylase/hydrolase 43* (*XTH43*) (McNeece et al., 2019). Each of these genes function effectively in the defense response that *G. max* has toward *H. glycines* (Matthews et al., 2013; Pant et al., 2014; Klink et al., 2017). In the case of XTH43, it functions to shorten xyloglucan chains, create more of those shorter chains, and make more xyloglucan (Niraula et al., 2021). The result provides actual biochemical support to the histological and ultrastructural observations that the cell walls do not expand during the defense response which normally happens during a susceptible response (Ross, 1958; Endo and Veech, 1970; Gipson et al., 1971; Riggs et al., 1973; Endo, 1991; Niraula et al., 2021).

**Figure 7:**
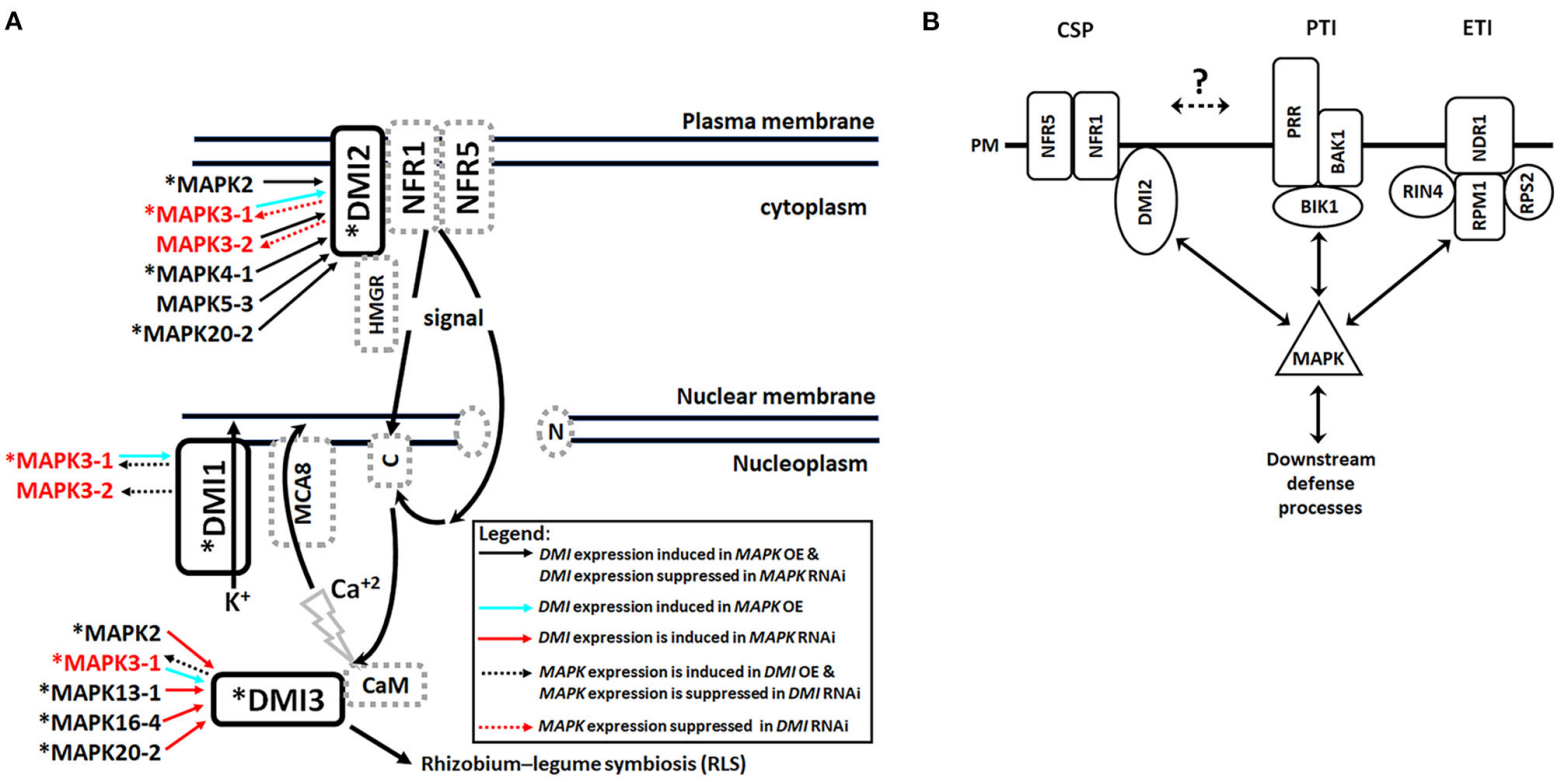
Model. **(A)** The plasma membrane (PM) receptor DMI2 which interacts with HMG-CoA reductase (3-hydroxy-3-methyl-glutaryl-coenzyme A reductase) (HMGR), and Nod factor receptors (NFR1 and NFR5), propagate signals that migrate either directly through the nuclear membrane or through nucleoporins (N), leading to the generation of calcium waves that involve the Type IIA calcium ATPase MCA8, and necessary to activate calmodulin and DMI3. The process concludes with an effective *Rhizobium*-legume symbiosis (RLS). The signaling process involves the engagement of the potassium channel DMI1. RNA-seq analyses, confirmed by RT-qPCR experiments, highlighted that some of the *MAPK*s undergoing transgenically-altered expression affects *DMI* RTAs as presented as their FC. Some of the involved MAPKs were determined to be expressed in syncytia undergoing the defense response (asterisk [*]) (McNeece et al., 2019). *expressed in syncytia undergoing a defense response. (adapted from Genre and Russo, 2016). **(B)** The *G. max* CSP, PTI, and ETI pathways converge on MAPK signaling, leading to downstream processes that function in defense to *H. glycines* parasitism. The hashed arrow between the CSP and PTI and ETI receptors is positioned because questions remain whether they directly influence their relative abundances (?).

The results presented here demonstrate that crosstalk occurs between the DMI-containing CSP and MAPK pathways. These results are significant because they point to the possible interaction occurring between the CSP and defense signaling, two ancient pathways found in land plants, and it is already known that PTI and ETI function through MAPKs in the *G. max*-*H. glycines* pathosystem (Figure 7). The results provide a genetic basis to further examine the CSP. Functional experiments aimed at examining all of the *DMI1, DMI2*, and *DMI3* paralogs would provide greater clarity on the roles of these genes in these processes. Determining whether DMI2 functions analogously as a co-receptor for NFR1 and NFR5 which bind NFs much like BAK1 does to various PRRs including FLAGELLIN-SENSITIVE 2 (FLS2), EF-Tu RECEPTOR (EFR) and the DAMP PEPTIDE 1 RECEPTOR (At-PEPR1) during defense processes is of interest (Li and Chory, 1997; Veronese et al., 2006; Zipfel et al., 2006; Chinchilla et al., 2007; Zhang et al., 2010; Liu et al., 2013). Like what has already been shown for *H. glycines* defense in *G. max*, an examination of other genes in the nodulation or CSP pathways would allow for greater resolution in understanding the defense process (Klink et al., 2021a). Lastly, transcriptomic analyses including RNA-seq of *DMI1, DMI2*, and *DMI3* OE and RNAi roots may also provide greater resolution in understanding the intricacies of the roles these genes play in the defense process. The results are consistent with experiments that show symbiosis-inducing organisms (AM) are capable of propagating Ca^2+^ spikes in *G. max* while also inducing the expression of heterologously expressed *M. truncatula DMI1, DMI2*, and *DMI3* (Navazio et al., 2007). Recent experiments show a reduction of 59-81% in cysts in AM-inoculated soybean, consistent with the results presented here where molecular context is provided (Pant et al., 2014; Aljaafri et al., 2017; McNeece et al., 2017, 2019; Pawlowski and Hartman, 2020; Klink et al., 2021a). An examination of other CSP genes and those that are not as well-conserved will provide additional, needed detail. Both defense and symbiosis processes involve the generation of Ca^2+^ signaling with defense involving cytoplasmic signals and symbiosis involving nuclear signals. The results presented here indicate the function and impact of the CSP regarding the interconnectedness of symbiosis and defense is far greater that previously appreciated. Part of its impact will be generating symbiotic interactions and defense processes where they currently do not exist or are not effective. Consequently, a functional transgenic examination of all *DMI1-3* paralogs and additional CSP genes in relation to defense and symbiosis are of interest.

## Data Availability Statement

The datasets presented in this study can be found in online repositories. The names of the repository/repositories and accession number(s) can be found at: National Center for Biotechnology Information (NCBI) BioProject database under accession number PRJNA664992.

## Author Contributions

RK, NA, and VK designed experiments, performed experiments, ran analyses, and wrote manuscript. SP, KS, PN, BL, and KL performed experiments, ran analyses, and wrote manuscript. All authors contributed to the article and approved the submitted version.

## Funding

VK: Cotton Incorporated (https://www.cottoninc.com/), grants 17-603, 19-603; Special Research Initiative, College of Arts and Sciences, Mississippi State University. KL: Hatch number: ALA015-2-14003. Mississippi Agricultural and Forestry Experiment Station (MAFES)-Special Research Initiative (SRI) grant.

## Conflict of Interest

The authors declare that the research was conducted in the absence of any commercial or financial relationships that could be construed as a potential conflict of interest.

## Publisher's Note

All claims expressed in this article are solely those of the authors and do not necessarily represent those of their affiliated organizations, or those of the publisher, the editors and the reviewers. Any product that may be evaluated in this article, or claim that may be made by its manufacturer, is not guaranteed or endorsed by the publisher.

## Supplementary Material

Supplementary Figure 1 Transgenic roots obtained from 6 dpi *H. glycines*-infected chimeric plants having genetically engineered roots and un-engineered shoots that were generated through the K599-mediated genetic transformation procedure. Please refer to Materials and Methods subsections: *DMI* gene cloning, and *G. max* genetic transformations, for details. FC was calculated by 2^−ΔΔCT^ (Livak and Schmittgen, 2001; Klink et al., 2021a). The RTA of the candidate defense genes, presented as a FC, in the transgenic roots was compared using the *G. max* ribosomal protein gene *RPS21* (Glyma.15G147700). The RT-qPCR analyses examined 3 experimental replicates (individual root systems) of *DMI1-3, DMI2-7*, and *DMI3-2* -OE or -RNAi roots as compared to their pRAP15-*ccd*B, and pRAP17-*ccd*B controls, respectively, from each of the 3 biological replicates. Each experimental replicate was run in triplicate using the same RNA. (^*^), statistical significance of *p* < 0.05, Student's *t*-test (Yuan et al., 2006). Please refer to Materials and Methods subsection: Real-time quantitative PCR (RT-qPCR), for details.

Supplementary Table 1 PCR, and RT-qPCR primers used in the analysis.

Supplementary Table 2 Raw *DMI* DCM data. Each experiment was replicated independently 3 times. Consequently, each experiment included three independent biological replicates. The cell (pericycle control, or syncytium) samples, obtained at different times have been presented as their respective time point (dpi). The three independent biological replicates included RNA samples isolated from the collected cells for each uninfected control (0 dpi), and *H. glycines*-infected (3, and 6 dpi) sample for each *G. max* genotype (*G*.*max*_[Peking/PI548402]_, and *G*.*max*_[PI88788]_). The replicated experiments, enacted at different times, were performed using three different microarrays (arrays) per *H. glycines*-resistant *G. max* genotype. These *H. glycines*-resistant *G. max* genotypes included *G*.*max*_[Peking/PI548402]_, and *G*.*max*_[PI88788]_. Red, gene expression was *measured* (M); blue, gene expression was *not measured* (NM); gray, *not applicable* (n/a) because no probe set existed on the microarray to measure expression for that gene (Klink et al., 2010a). The DCM analysis that led to the results occurred using data derived from the three independent replicates with a statistical significance threshold of *p* < 0.05, Mann–Whitney–Wilcoxon (MWW) Rank Sum Test (Mann and Whitney, 1947). The genes highlighted in yellow were studied subsequently in the functional transgenic experiments (presented in Figures 1, 2). Accession 1: Wm82.a1.v1.1 (2010) annotation. Annotation 2: Wm82.a2.v1 (2015) annotation. Please refer to Materials and Methods subsection: The identification of *DMI* gene families and root cell-specific expression, for further details.

Supplementary Table 3 Summarized *G. max DMI* genes expressed in syncytia. Gene families and syncytium expression profiles were presented in Supplementary Table 2. The DMI protein family members were identified from the *G. max* genome through conceptual translation of their gene sequences according to the procedures outlined in the Materials and Methods subsection: Selection of candidate genes. The accessions had represented the gene identifier obtained from the *G. max* genome. The Accession 1 identifier was obtained from the later 2015 (Wm82.a2.v1) annotation. The Accession 2 identifier was obtained from the earlier 2010 (Wm82.a1.v1.1) annotation. Annotation 2 relates directly to the annotation used in the Klink et al. (2010a,b) Affymetrix® gene expression study employed to generate the gene expression data for the syncytium undergoing the defense process. Table header description: *Affymetrix® p/s*, probe set identifier relating to the probe set that was fabricated onto the microarray and used to generate the gene expression data. *Annotation*, the *G. max* genome annotation of the gene. *Time points*, the time point (dpi), of *H. glycines* infection at which time the *G. max* root cells was collected by LM with their isolated RNA used for the gene expression study. Homologs identified to be expressed in syncytia undergoing the defense response, and not in control cells, studied in the functional transgenic analyses was highlighted in yellow. Blue, expression *not measured* (NM) to statistically significant levels, *p* ≥ 0.05. Red, expression *measured* (M) to statistically significant level, *p* < 0.05. The statistical significance (^*^) was calculated using the Mann–Whitney–Wilcoxon (MWW) Rank-Sum Test, *p* < 0.05 (Mann and Whitney, 1947). Please refer to Materials and Methods subsection: The identification of *DMI* gene families and root cell-specific expression, for details.

Supplementary Table 4 DMI1 homologs that exist in select agriculturally important crops (Tilman et al., 2011; Ray et al., 2013, 2019). Values not in parentheses have a Blast of e-0. Numbers in parentheses have a Blast cutoff of > e-0. Please refer to Materials and Methods subsections: *G. max DMI* gene identification, and Proteome mining, for details.

Supplementary Table 5 DMI2 homologs that exist in select agriculturally important crops (Tilman et al., 2011; Ray et al., 2013, 2019). Values not in parentheses have a Blast of e-0. Numbers in parentheses have a Blast cutoff of > e-0. Please refer to Materials and Methods subsections: *G. max DMI* gene identification, and Proteome mining, for details.

Supplementary Table 6 DMI3 homologs that exist in agriculturally important crops (Tilman et al., 2011; Ray et al., 2013, 2019). Values not in parentheses have a Blast of e-0. Numbers in parentheses have a Blast cutoff of > e-0. Please refer to Materials and Methods subsections: *G. max DMI* gene identification, and Proteome mining, for details.

Supplementary Table 7 *DMI* gene expression in defense *MAPK*-overexpressing (*MAPK*-OE) root RNA as compared to the pRAP15-*ccd*B control root RNA. RNA-seq data whose differential expression is presented as an RTA that represents FC; obtained from Alshehri et al. (2019) (*p* < 0.001) (Wang and Wang, 2021). Please refer to Materials and Methods subsection: RNA-seq gene expression analyses of transgenic *MAPK*-OE and **-**RNAi root RNA, for details.

Supplementary Table 8 *DMI* gene expression in defense *MAPK*-RNA interference (*MAPK*-RNAi) root RNA as compared to the pRAP17-*ccd*B control root RNA. RNA-seq data whose differential expression was calculated as an RTA has been presented as FC; obtained from Alshehri et al. (2019) (*p* < 0.001) (Wang and Wang, 2021). Please refer to Materials and Methods subsection: RNA-seq gene expression analyses of transgenic *MAPK*-OE and **-**RNAi root RNA, for details.
