# Supplementary figures and images for: Glycine max Homologs of DOESN'T MAKE INFECTIONS 1, 2, and 3 Function to Impair Heterodera glycines Parasitism While Also Regulating Mitogen Activated Protein Kinase Expression
Source: Front Plant Sci. 2022 May 4;13:842597. doi: 10.3389/fpls.2022.842597 (PMC9114929; doi:10.3389/fpls.2022.842597)

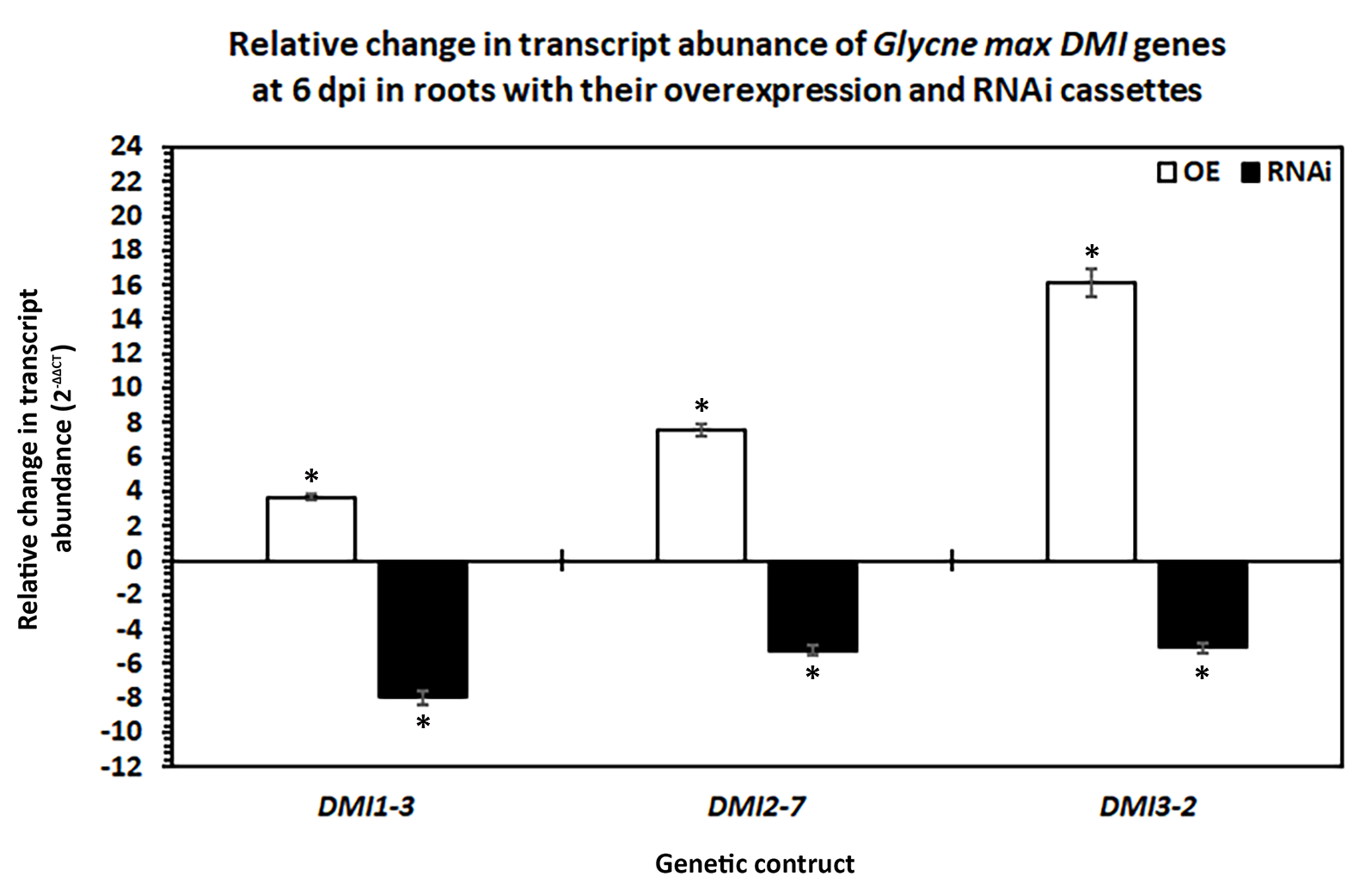

Supplement: Supplementary Figure 1 — Transgenic roots obtained from 6 dpi H. glycines-infected chimeric plants having genetically engineered roots and un-engineered shoots that were generated through the K599-mediated genetic transformation procedure. Please refer to Materials and Methods subsections: DMI gene cloning, and G. max genetic transformations, for details. FC was calculated by 2−ΔΔCT (Livak and Schmittgen, 2001; Klink et al., 2021a). The RTA of the candidate defense genes, presented as a FC, in the transgenic roots was compared using the G. max ribosomal protein gene RPS21 (Glyma.15G147700). The RT-qPCR analyses examined 3 experimental replicates (individual root systems) of DMI1-3, DMI2-7, and DMI3-2 -OE or -RNAi roots as compared to their pRAP15-ccdB, and pRAP17-ccdB controls, respectively, from each of the 3 biological replicates. Each experimental replicate was run in triplicate using the same RNA. (*), statistical significance of p < 0.05, Student's t-test (Yuan et al., 2006). Please refer to Materials and Methods subsection: Real-time quantitative PCR (RT-qPCR), for details. [file Image_1.TIF]
